# Polyphosphoric Acid-Promoted Efficient Synthesis of Cinnamides via Aldol Condensation of Amide

**DOI:** 10.3390/molecules29194632

**Published:** 2024-09-29

**Authors:** Enhua Wang, Lishou Yang, Lanfeng He, Qian Yang, Xue Wang, Yunlu Liu, Manxiang Li, Yang Lei, Xiaosheng Yang

**Affiliations:** 1 Department of Food and Medicine, Guizhou Vocational College of Agriculture, Qingzhen 551400, China; enhuawang2023@sina.cn (E.W.); yangqian121212@sina.cn (Q.Y.); wangxue155@sina.com (X.W.); liuyunlu12@sina.com (Y.L.); limanxiang1224@sina.com (M.L.); 2 State Key Laboratory of Functions and Applications of Medicinal Plants, Guizhou Medical University, Guiyang 550014, China; lishouyang2023@sina.cn (L.Y.); yao320917547@sina.com (L.H.); 3 Natural Products Research Center of Guizhou Province, Guiyang 550014, China

**Keywords:** polyphosphoric acid, cinnamide, aldol condensation, amide

## Abstract

Cinnamides are common core structures that exist in a great number of pharmaceuticals and natural products. The development of efficient methods for preparing cinnamides is in great need. We report herein an efficient polyphosphoric acid (PPA)-promoted direct aldol condensation of an amide for the convenient and straightforward preparation of cinnamides. A variety of cinnamides were obtained in moderate-to-excellent yields (65–89%). This strategy features the use of equivalent amides and a short reaction time.

## 1. Introduction

**Figure 1:**
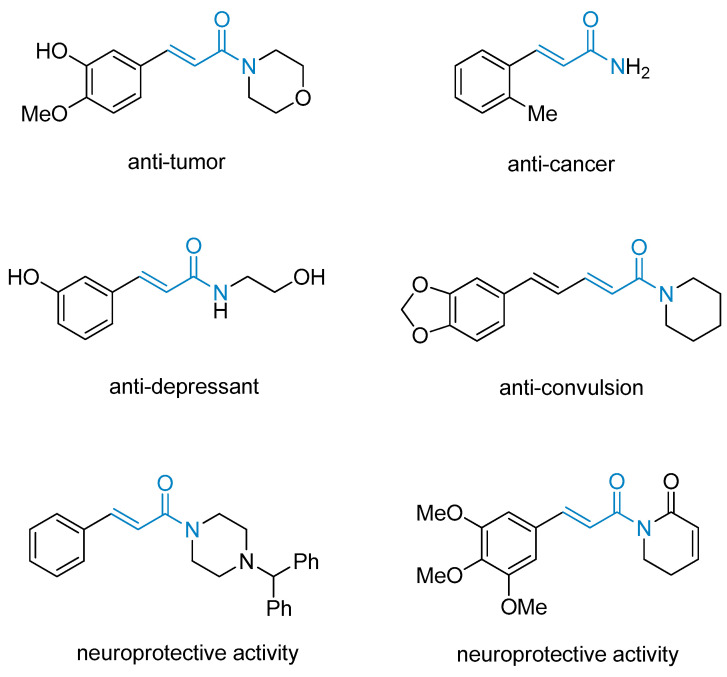
Selected bioactive cinnamide derivatives.

Cinnamides have been used as building blocks for the construction of complex molecules, which commonly exist in pharmaceuticals and natural products [1,2,3,4,5,6]. Most of the cinnamides display varied bioactivities such as anti-tumor, anti-cancer, anti-depressant, and anti-convulsion effects (Figure 1) [7,8,9,10]. Very recently, Wu and co-workers reported that cinnamide derivatives exhibited neuroprotective activity and have potential for the treatment of cerebral ischemia [11,12]. Biersack and co-workers found that piperlongumine-type cinnamide derivatives showed good antiparasitic and antifungal activities [13]. Therefore, the development of efficient methods for preparing cinnamides is still a hot research topic in the field of organic chemistry. Commonly, cinnamides are synthesized from the acylation of amines with cinnamic acids [14,15,16], decarboxylative acylation of cinnamic acids [17], C-H bond functionalizations [18,19,20,21,22,23,24], and amidation of carboxylic acids [25]. However, the above-mentioned reactions suffer from limitations such as reliance on multisteps, the use of strong oxidants and special substrates, and catalyzation by transition metal or toxic metal salts.

**Scheme 1:**
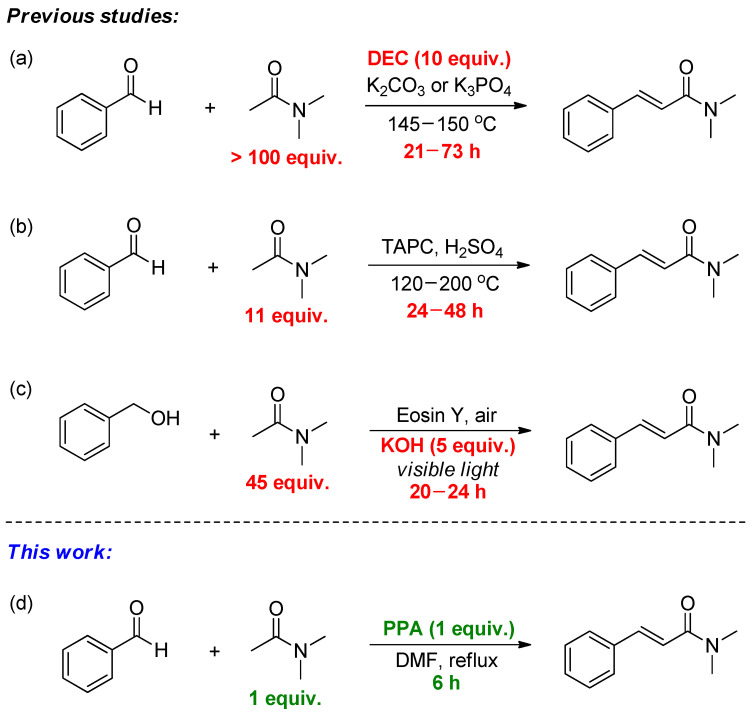
Previous reported strategies for the synthesis of cinnamides and this work. (**a**) Previous work: DEC-promoted aldol condensation of unactivated amides for the synthesis of cinnamides. (**b**) Previous work: TAPC and H_2_SO_4_ catalyzed aldol condensation of unactivated amides for the synthesis of cinnamides. (**c**) Visible light-promoted oxidation/condensation of benzyl alcohols with amides to cinnamides. (**d**) This work: PPA-promoted direct aldol condensation of unactivated amides for the convenient and straightforward synthesis of cinnamides.

Considering the limitations of the above approaches, the convenient preparations of cinnamides are highly desired. In 2010, the direct aldol condensation of unactivated amides catalyzed by dialkylcarbonates (DECs), as dehydrating agents, was reported (Scheme 1a) [26]. Later, Saito and co-workers reported a similar strategy for the preparation of cinnamides using 1,3,5-triazo-2,4,6-triphosphorine-2,2,4,4,6,6-hexachloride-based phosphorous (TAPC)/SO_4_^2−^ catalysis (Scheme 1b) [27]. Despite significant progress, the lowest acidity of the α-CH of amides results in major shortcomings such as the excessive use of dehydrating agents and amides, and the requirement of long reaction times (Scheme 1a,b). Recently, Cai and co-workers developed a visible light-promoted oxidative cross-coupling reaction of benzyl alcohols with *N*,*N*-dialkylacetamides for the construction of cinnamides; however, this strategy still needs excessive base and amides, as well as a long reaction time (Scheme 1c) [28]. Hence, the development of new synthetic strategies devoid of these disadvantages is still urgent.

The direct aldol condensation of unactivated amides is a straightforward and attractive methodology, but there is a need to develop an effective dehydrating agent to overcome the above disadvantages. PPA is commonly used as a dehydrating agent, and our group has developed several PPA-promoted condensation reactions for the synthesis of heterocycles [29,30,31]. Our previous studies indicated that PPA could activate the α-CH of the carbonyl group and efficiently promote aldol condensations. Inspired by our previous work and in continuation of our interest in PPA-promoted condensations, herein we wish to describe another direct aldol condensation of unactivated amides using PPA as an activator and dehydrating agent for the convenient and straightforward synthesis of cinnamides (Scheme 1d). This method features the use of equivalent amides and a short reaction time.

## 2. Results and Discussion

Initially, we set up the reaction benzaldehyde **1a** and *N*,*N*-dimethylacetamide **2** as test substrates. A mixture of **1a** (1 equiv.), **2** (1 equiv.), and PPA (2 equiv.) in DMF refluxed for 4 h under a nitrogen atmosphere produced the cinnamide **3a** in a 46% yield (Table 1, entry 1). Screening of the amount of PPA showed that 1 equiv. was the best choice (Table 1, entries 2–4). Then, we investigated the influence of reaction temperature, and a decreased yield was observed upon decreasing the temperature to 140 °C (Table 1, entry 5 vs. 3). Subsequently, we tested the effect of the reaction time (Table 1, entries 6–8). To our delight, the reaction was found to be more effective when the reaction time increased to 6 h, which gave the desired product in a 79% yield (Table 1, entry 7). Next, we screened a serious of reaction media, such as 1,4-dioxane, THF, toluene, DCE, DMSO, and EtOH, and no improvement in the transformation was observed (Table 1, entries 9–14). Finally, the optimized reaction conditions were determined as **1a** (1 equiv.), **2** (1 equiv.), and PPA (1 equiv.) in DMF refluxed for 6 h under a nitrogen atmosphere (Table 1, entry 7).

**Scheme 2:**
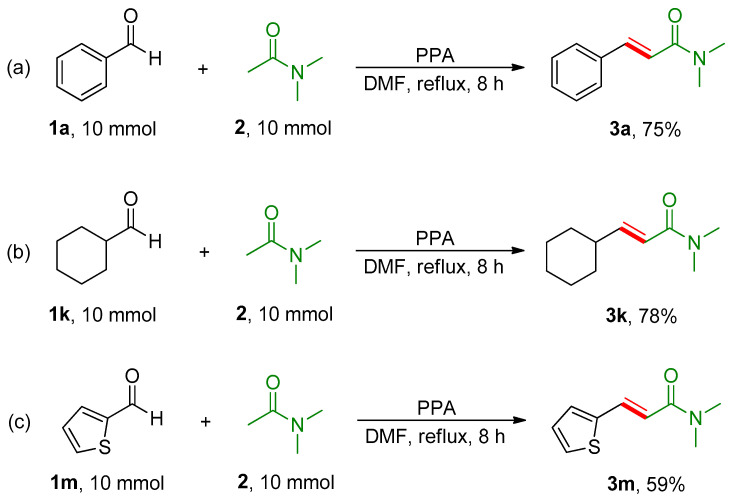
Gram-scale reactions. Gram-scale reactions of benzaldehyde **1a** (**a**), cyclohexanecarboxaldehyde **1k** (**b**), and 2-thenaldehyde **1m** (**c**). Reaction conditions: PPA (10 mmol) and DMF (10 mL).

With the optimized reaction conditions in hand, we next examined the scope of aldehydes (Table 2). As shown in Table 2, the condensation proceeded well with a variety of benzaldehydes to yield desired products in moderate-to-good yields (**3a**–**3i**). Obviously, the electronic properties of the substituents had an effect on the yield. Benzaldehydes bearing electron-donating groups (OMe and Me) provided higher yields than those bearing electron-withdrawing groups, such as F, Cl, Br, CF_3_, and CN (**3b**–**3g** vs. **3h**–**3i**). It was also found that this procedure was efficient for converting 2-naphthaldehyde, cyclohexanecarboxaldehyde, valeraldehyde, 2-thenaldehyde, 4-benzofurazancarboxaldehyde, and cinnamaldehyde to the corresponding cinnamides in good yields (**3j**–**3o**). Next, to demonstrate the synthetic potential of this protocol, we selected different types of aldehydes for gram-scale reactions (Scheme 2). To our delight, the gram-scale reactions also proceeded well to form the corresponding products in good yields.

**Scheme 3:**
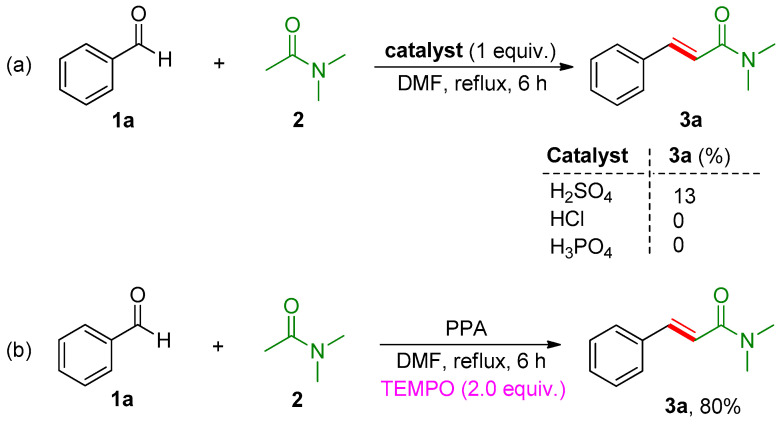
Control experiments. (**a**) Effect of catalyst on the reaction. (**b**) Investigation of free radical mechanism. Reaction conditions: **1a** (1 mmol), **2** (1 mmol), PPA (1 mmol), catalyst (1 mmol), and DMF (1 mL).

Several control experiments were conducted in order to rationalize the reaction mechanism. As shown in Scheme 3a, the reaction was carried out in the presence of different dehydrating agent/acid catalysts and the desired product **3a** was obtained in a 13% yield only in the case of using concentrated sulfuric acid, which demonstrated that this reaction underwent a dehydrating process. Then, the radical inhibitor 2,2,6,6-tetramethyl-piperidine-1-oxyl (TEMPO) was employed in the reaction, yielding **3a** in a high yield, suggesting that this procedure rules out the radical pathway (Scheme 3b).

**Scheme 4:**
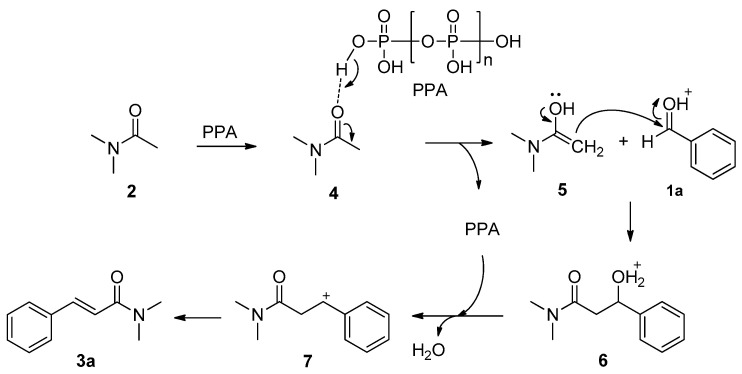
Plausible mechanism.

Based on the mechanistic study and our previous reports [29,32], a plausible mechanism of the PPA-promoted aldol condensation of amides for the synthesis of cinnamides is depicted in Scheme 4. Firstly, amide **2** was activated through the formation of the phosphorylated adduct **4**, followed by an enolization to produce intermediate **5**. Then, intermediate **5** coupled with protonated benzaldehyde **1a** formed intermediate **6**, which was followed by a PPA-driven dehydration process to give intermediate **7**. Finally, the carbocation is stabilized by removing a proton from the beta position yielding the desired product **3a**.

## 3. Materials and Methods

### 3.1. General Information

All reagents, catalysts, and solvents were purchased from commercial suppliers and used without further purification unless otherwise noted. Column chromatography was carried out with silica gel (200–300 mesh). An X-4 melting point apparatus with a microscope (Dedu Precision Instrument Co., Ltd, Changzhou, China) was used for melting point determination. The IR spectra were recorded with Mattson FTIR spectrometer 5000 (Mattson, MA, USA). Absorption maxima were measured in cm^−1^. ^1^H NMR (600 MHz) and ^13^C NMR (150 MHz) spectra were achieved in CDCl_3_ on a Bruker AVANCE 600 MHz spectrometer (Bruker, Saarbrücken, Germany). High-resolution mass spectra were measured on a ThermoFisher QE Focus facility (Thermo Fisher Scientific, Waltham, MA, USA). The copies of the ^1^H NMR and ^13^C NMR spectra for all compounds see Supplementary Materials.

### 3.2. General Procedure for the Synthesis of Cinnamamides ***3a***–***3o***

Benzaldehyde (1 mmol), DMA (1 mmol), and PPA (1 mmol) were added to a 5 mL round-bottomed flask containing DMF (1 mL) solvent. Under the protection of nitrogen, the reaction mixture was refluxed for 6 h. The reaction liquid was quenched with water and extracted with ethyl acetate. The organic layer was dried by anhydrous Na_2_SO_4_ and evaporated under reduced pressure. The resulting crude compound was purified by silica gel column chromatography to yield the pure products **3a**–**3o**.

*(E)-N,N-Dimethylcinnamamide* (**3a**). White solid; yield: 79%; m.p.: 100–104 °C. IR (KBr plate): *ν*_max_ 1654 (C=O), 1648 (C=C), 1141 (C-N), 766 (Ar-H). ^1^H NMR (600 MHz, CDCl_3_) δ 7.67–7.63 (dd, *J* = 15.4, 2.1 Hz, 1H, Ar-CH=), 7.52–7.49 (m, 2H, Ar-H), 7.36–7.32 (m, 3H, Ar-H), 6.89–6.86 (dd, *J* = 15.4, 2.1 Hz, 1H, CO-CH=), 3.14 (s, 3H, CH_3_), 3.04 (s, 3H, CH_3_). ^13^C NMR (150 MHz, CDCl_3_) δ 166.7 (C=O), 142.3 (-CH=), 135.4 (Ar-C), 129.6 (Ar-C), 128.8 (Ar-C), 127.8 (Ar-C), 117.4 (-CH=), 37.4 (CH_3_), 35.9 (CH_3_). HRMS-ESI (*m*/*z*): calcd. for C_11_H_13_ONNa [M + Na]^+^: 198.0889; found: 198.0887.
*(E)-3-(2-Fluorophenyl)-N,N-dimethylacrylamide* (**3b**). White solid; yield: 78%; m.p.: 58–61 °C. IR (KBr plate): *ν*_max_ 2941 (C-H), 1654 (C=O), 1597 (C=C), 1143 (C-N), 991 (Ar-H), 755 (Ar-H). ^1^H NMR (600 MHz, CDCl_3_) δ 7.70 (dd, *J* = 15.8, 3.1 Hz, 1H, Ar-CH=), 7.49 (q, *J* = 6.3, 5.5 Hz, 1H, Ar-H), 7.31–7.26 (m, 1H, Ar-H), 7.12 (d, *J* = 7.3 Hz, 1H, Ar-H), 7.09–6.99 (m, 2H, Ar-H, CO-CH=), 3.14 (s, 3H, CH_3_), 3.05 (s, 3H, CH_3_). ^13^C NMR (150 MHz, CDCl_3_) δ 166.6 (C=O), 161.3 (*^1^J_CF_* = 253.7, Ar-C), 135.3 (-CH=), 130.8 (*^3^J_CF_* = 9.1, Ar-C), 129.8, 124.3 (*^3^J_CF_* = 3.0, Ar-C), 123.3 (*^2^J_CF_* = 12.1, Ar-C), 120.5 (*^3^J_CF_* = 7.6, -CH=), 116.1 (*^2^J_CF_* = 21.1, Ar-C), 37.4 (CH_3_), 35.9 (CH_3_). HRMS-ESI (*m*/*z*): calcd. for C_11_H_12_ONF [M + Na]^+^: 216.0795; found: 216.0794.
*(E)-3-(2-Chlorophenyl)-N,N-dimethylacrylamide* (**3c**). White solid; yield: 80%; m.p.: 80–84 °C. IR (KBr plate): *ν*_max_ 1648 (C=O), 1610 (C=C), 1143 (C-N), 759 (Ar-H). ^1^H NMR (600 MHz, CDCl_3_) δ 7.99 (d, *J* = 15.5 Hz, 1H, Ar-CH=), 7.59–7.57 (m, 1H, Ar-H), 7.40–7.36 (m, 1H, Ar-H), 7.26–7.23 (m, 2H, Ar-H), 6.86 (d, *J* = 15.5 Hz, 1H, CO-CH=), 3.15 (s, 3H, CH_3_), 3.05 (s, 3H, CH_3_). ^13^C NMR (150 MHz, CDCl_3_) δ 166.3 (C=O), 138.2 (-CH=), 134.6 (Ar-C), 133.7 (Ar-C), 130.3 (Ar-C), 130.1 (Ar-C), 127.7 (Ar-C), 126.9 (Ar-C), 120.6 (-CH=), 37.5 (CH_3_), 35.9 (CH_3_). HRMS-ESI (*m*/*z*): calcd. for C_11_H_12_NOClNa [M + Na]^+^: 232.0500; found: 232.0498.
*(E)-3-(2,4-Dichlorophenyl)-N,N-dimethylacrylamide* (**3d**). White solid; yield: 67%; m.p.: 138–141 °C. IR (KBr plate): *ν*_max_ 2935 (C-H), 1650 (C=O), 1602 (C=C), 1141 (C-N), 768 (Ar-H). ^1^H NMR (600 MHz, CDCl_3_) δ 7.93 (d, *J* = 15.4 Hz, 1H, Ar-CH=), 7.53 (d, *J* = 8.4 Hz, 1H, Ar-H), 7.42 (d, *J* = 2.1 Hz, 1H, Ar-H), 7.24 (dd, *J* = 8.4, 2.1 Hz, 1H, Ar-H), 6.86 (d, *J* = 15.4 Hz, 1H, CO-CH=), 3.17 (s, 3H, CH_3_), 3.07 (s, 3H, CH_3_). ^13^C NMR (150 MHz, CDCl_3_) δ 166.1 (C=O), 137.1 (-CH=), 135.4 (Ar-C), 135.2 (Ar-C), 132.4 (Ar-C), 129.9 (Ar-C), 128.4 (Ar-C), 127.4 (Ar-C), 121.0 (-CH=), 37.5 (CH_3_), 36.0 (CH_3_). HRMS-ESI (*m*/*z*): calcd. for C_11_H_11_ONCl_2_Na [M + Na]^+^: 266.0110; found: 266.0108.
*(E)-3-(4-Bromophenyl)-N,N-dimethylacrylamide* (**3e**). White solid; yield: 69%; m.p.: 127–130 °C. IR (KBr plate): *ν*_max_ 1648 (C=O), 1595 (C=C), 1147 (C-N), 751 (Ar-H). ^1^H NMR (600 MHz, CDCl_3_) δ 7.59 (d, *J* = 15.4 Hz, 1H, Ar-CH=), 7.49 (d, *J* = 8.3 Hz, 2H, Ar-H), 7.38 (d, *J* = 8.4 Hz, 2H, Ar-H), 6.87 (d, *J* = 15.4 Hz, 1H, CO-CH=), 3.16 (s, 3H, CH_3_), 3.06 (s, 3H, CH_3_). ^13^C NMR (150 MHz, CDCl_3_) δ 166.4 (C=O), 141.1 (-CH=), 134.3 (Ar-C), 132.0 (Ar-C), 129.2 (Ar-C), 123.6 (Ar-C), 118.1 (-CH=), 37.4 (CH_3_), 36.0 (CH_3_). HRMS-ESI (*m*/*z*): calcd. for C_11_H_13_ONBr [M + H]^+^: 254.0175; found: 254.0173.
*(E)-N,N-Dimethyl-3-(4-(trifluoromethyl)phenyl)acrylamide* (**3f**). White solid; yield: 66%; m.p.: 110–112 °C. IR (KBr plate): *ν*_max_ 2935 (C-H), 1656 (C=O), 1607 (C=C), 818 (Ar-H). ^1^H NMR (600 MHz, CDCl_3_) δ 7.63–7.57 (m, 1H, Ar-CH=), 7.56–7.50 (m, 4H, Ar-H), 6.92 (dd, *J* = 15.5, 3.7 Hz, 1H, CO-CH=), 3.13–3.08 (m, 3H, CH_3_), 3.02–2.97 (m, 3H, CH_3_). ^13^C NMR (150 MHz, CDCl_3_) δ 166.0 (C=O), 140.5 (-CH=), 138.7 (Ar-C), 130.9(*^2^J_CF_* = 30.2, Ar-C), 127.9 (Ar-C), 125.6 (Ar-C), 123.9 (*^1^_CF_* = 271.8, CF_3_), 120.0 (-CH=), 37.3 (CH_3_), 35.9 (CH_3_). HRMS-ESI (*m*/*z*): calcd. for C_12_H_12_ONF_3_Na [M + Na]^+^: 266.0763; found: 266.0762.
*(E)-3-(4-Cyanophenyl)-N,N-dimethylacrylamide* (**3g**). White solid; yield: 77%; m.p.: 149–154 °C. IR (KBr plate): *ν*_max_ 1650 (C=O), 1608 (C=C), 1143 (C-N), 834 (Ar-H), 824 (Ar-H). ^1^H NMR (600 MHz, CDCl_3_) δ 7.63 (m, 2H, Ar-CH=, Ar-H), 7.58 (d, *J* = 8.1 Hz, 3H, Ar-H), 6.96 (d, *J* = 15.4 Hz, 1H, CO-CH=), 3.16 (d, *J* = 4.8 Hz, 3H, CH_3_), 3.05–3.03 (m, 3H, CH_3_). ^13^C NMR (150 MHz, CDCl_3_) δ 165.8 (C=O), 140.1 (-CH=), 139.7 (Ar-C), 132.6 (Ar-C), 128.20 (Ar-C), 121.1 (CN), 118.6 (-CH=), 112.6 (Ar-C), 37.5 (CH_3_), 36.0 (CH_3_). HRMS-ESI (*m*/*z*): calcd. for C_12_H_12_ON_2_Na [M + Na]^+^: 223.0842; found: 223.0840.
*(E)-N,N-Dimethyl-3-(3-phenoxyphenyl)acrylamide* (**3h**). White solid; yield: 81%; m.p.: 71–73 °C. IR (KBr plate): *ν*_max_ 3024 (C-H), 2927 (C-H), 1651 (C=O), 1607 (C=C), 1164 (C-N), 754 (Ar-H). ^1^H NMR (600 MHz, CDCl_3_) δ 7.62 (d, *J* = 15.4 Hz, 1H, Ar-CH=), 7.35 (dt, *J* = 14.8, 7.9 Hz, 3H, Ar-H), 7.27 (d, *J* = 7.8 Hz, 1H, Ar-H), 7.20 (s, 1H, Ar-H), 7.13 (t, *J* = 7.4 Hz, 1H, Ar-H), 7.03 (d, *J* = 8.4 Hz, 2H, Ar-H), 6.99 (dd, *J* = 8.1, 2.5 Hz, 1H, Ar-H), 6.86 (d, *J* = 15.4 Hz, 1H, CO-CH=), 3.16 (s, 3H, CH_3_), 3.07 (s, 3H, CH_3_). ^13^C NMR (150 MHz, CDCl_3_) δ 166.5 (C=O), 157.6 (Ar-C), 156.9 (Ar-C), 141.7 (-CH=), 137.2 (Ar-C), 130.1 (Ar-C), 129.9 (Ar-C), 123.5 (Ar-C), 123.0 (Ar-C), 119.8 (Ar-C), 118.9 (Ar-C), 118.2 (-CH=), 117.7 (Ar-C), 37.5 (CH_3_), 36.0 (CH_3_). HRMS-ESI (*m*/*z*): calcd. for C_17_H_17_O_2_NNa [M + Na]^+^: 290.1152; found: 290.1148.
*(E)-N,N-Dimethyl-3-(p-tolyl)acrylamide* (**3i**). White solid; yield: 85%; m.p.: 112–117 °C. IR (KBr plate): *ν*_max_ 2935 (C-H), 1650 (C=O), 1602 (C=C), 1141 (C-N), 768 (Ar-H). ^1^H NMR (600 MHz, CDCl_3_) δ 7.64 (d, *J* = 15.4 Hz, 1H, Ar-CH=), 7.42 (d, *J* = 8.0 Hz, 2H, Ar-H), 7.17 (d, *J* = 7.8 Hz, 2H, Ar-H), 6.84 (d, *J* = 15.4 Hz, 1H, CO-CH=), 3.15 (s, 3H, CH_3_), 3.05 (s, 3H, CH_3_), 2.35 (s, 3H, Ar-CH_3_). ^13^C NMR (150 MHz, CDCl_3_) δ 166.9 (C=O), 142.3 (-CH=), 139.8 (Ar-C), 132.6 (Ar-C), 129.5 (Ar-C), 127.8 (Ar-C), 116.3 (-CH=), 37.4 (CH_3_), 35.9 (CH_3_), 21.4 (CH_3_). HRMS-ESI (*m*/*z*): calcd. for C_12_H_15_ONNa [M + Na]^+^: 212.1046; found: 212.1044.
*(E)-N,N-Dimethyl-3-(naphthalen-2-yl)acrylamide* (**3j**). Light yellow solid; yield: 78%; m.p.: 158–161 °C. IR (KBr plate): *ν*_max_ 2934 (C-H), 1648 (C=O), 1601 (C=C), 1138 (C-N), 822 (Ar-H). ^1^H NMR (600 MHz, CDCl_3_) δ 7.91 (s, 1H, Ar-H), 7.82 (m, 4H, Ar-CH=, Ar-H), 7.67 (d, *J* = 8.6 Hz, 1H, Ar-H), 7.48 (dd, *J* = 6.2, 3.2 Hz, 2H, Ar-H), 6.99 (d, *J* = 15.4 Hz, 1H, CO-CH=), 3.19 (s, 3H, CH_3_), 3.08 (s, 3H, CH_3_). ^13^C NMR (150 MHz, CDCl_3_) δ 166.6 (C=O), 142.4 (-CH=), 133.9 (Ar-C), 133.4 (Ar-C), 132.8 (Ar-C), 129.2 (Ar-C), 128.5 (Ar-C), 128.5 (Ar-C), 127.7 (Ar-C), 126.9 (Ar-C), 126.6 (Ar-C), 123.7 (Ar-C), 117.6 (-CH=), 37.5 (CH_3_), 36.0 (CH_3_). HRMS-ESI (*m*/*z*): calcd. for C_15_H_15_ONNa [M + Na]^+^: 248.1046; found: 248.1045.
*(E)-3-Cyclohexyl-N,N-dimethylacrylamide* (**3k**). Light yellow solid; yield: 89%; m.p.: 96–100 °C. IR (KBr plate): *ν*_max_ 1647 (C=O), 1602 (C=C), 1140 (C-N). ^1^H NMR (600 MHz, CDCl_3_) δ 6.80 (dd, *J* = 15.2, 7.0 Hz, 1H, Ar-CH=), 6.17 (d, *J* = 15.2 Hz, 1H, CO-CH=), 3.05 (s, 3H, CH_3_), 2.98 (s, 3H, CH_3_), 2.14–2.08 (m, 1H, Cy-H), 1.76–1.71 (m, 4H, Cy-H), 1.65 (m, 1H, Cy-H), 1.26 (m, 2H, Cy-H), 1.14 (m, 3H, Cy-H). ^13^C NMR (150 MHz, CDCl_3_) δ 167.2 (C=O), 151.3 (-CH=), 117.6 (-CH=), 40.7 (Cy-C), 37.3 (CH_3_), 35.7 (CH_3_), 32.0 (Cy-C), 25.9 (Cy-C), 25.7 (Cy-C). HRMS-ESI (*m*/*z*): calcd. for C_11_H_20_ON [M + H]^+^: 182.1539; found: 182.1539.
*(E)-N,N-Dimethylhept-2-enamide* (**3l**). White oil; yield: 74%; IR (KBr plate): *ν*_max_ 2938 (C-H), 1654 (C=O), 1611 (C=C), 1143 (C-N). ^1^H NMR (600 MHz, CDCl_3_) δ 6.88 (dt, *J* = 14.5, 7.0 Hz, 1H, Ar-CH=), 6.25 (d, *J* = 15.1 Hz, 1H, CO-CH=), 3.09 (s, 3H, CH_3_), 3.01 (s, 3H, CH_3_), 2.22 (q, *J* = 7.4 Hz, 2H, CH_2_), 1.46 (t, *J* = 7.7 Hz, 2H, CH_2_), 1.36 (q, *J* = 7.4 Hz, 2H, CH_2_), 0.92 (t, *J* = 7.3 Hz, 3H, CH_3_). ^13^C NMR (150 MHz, CDCl_3_) δ 167.0 (C=O), 146.4 (-CH=), 120.1 (-CH=), 37.4 (CH_3_), 35.7 (CH_3_), 32.2 (CH_2_), 30.5 (CH_2_), 22.3 (CH_2_), 13.9 (CH_3_). HRMS-ESI (*m*/*z*): calcd. for C_9_H_17_ONNa [M + Na]^+^: 178.1202; found: 178.1200.
*(E)-N,N-Dimethyl-3-(thiophen-2-yl)acrylamide* (**3m**). Light yellow solid; yield: 71%; m.p.: 98–101 °C. IR (KBr plate): *ν*_max_ 2930 (C-H), 1643 (C=O), 1601 (C=C), 1140 (C-N), 705 (Th-H). ^1^H NMR (600 MHz, CDCl_3_) δ 7.79 (d, *J* = 15.1 Hz, 1H, Ar-CH=), 7.31 (d, *J* = 5.1 Hz, 1H, CO-CH=), 7.22 (d, *J* = 3.6 Hz, 1H, Th-H), 7.03 (dd, *J* = 5.0, 3.6 Hz, 1H, Th-H), 6.69 (d, *J* = 15.1 Hz, 1H, Th-H), 3.15 (s, 3H, CH_3_), 3.06 (s, 3H, CH_3_). ^13^C NMR (150 MHz, CDCl_3_) δ 166.4 (C=O), 140.5 (-CH=), 135.2 (Th-C), 130.3 (Th-C), 128.0 (Th-C), 127.2 (Th-C), 116.1 (-CH=), 37.4 (CH_3_), 36.0 (CH_3_). HRMS-ESI (*m*/*z*): calcd. for C_9_H_11_ONNaS [M + Na]^+^: 204.0454; found: 204.0453.
*(E)-3-(Benzo[c][1,2,5]oxadiazol-4-yl)-N,N-dimethylacrylamide* (**3n**). Light yellow solid; yield: 65%; m.p.: 154–160 °C. IR (KBr plate): *ν*_max_ 2931 (C-H), 1651 (C=O), 1612 (C=C), 1143 (C-N), 812 (Ar-H), 766 (Ar-H). ^1^H NMR (600 MHz, CDCl_3_) δ 7.94 (d, *J* = 15.3 Hz, 1H, Ar-CH=), 7.82 (d, *J* = 8.6 Hz, 1H, Ar-H), 7.76 (d, *J* = 15.2 Hz, 1H, CO-CH=), 7.49–7.44 (m, 2H, Ar-H), 3.28 (s, 3H, CH_3_), 3.12 (s, 3H, CH_3_). ^13^C NMR (150 MHz, CDCl_3_) δ 166.2 (C=O), 149.6 (Ar-C), 147.5 (-CH=), 136.5 (Ar-C), 133.7 (Ar-C), 131.7 (Ar-C), 126.2 (Ar-C), 125.3 (Ar-C), 117.0 (-CH=), 37.5 (CH_3_), 36.0 (CH_3_). HRMS-ESI (*m*/*z*): calcd. for C_11_H_11_O_2_N_3_Na [M + Na]^+^: 240.0743; found: 240.0741.
*(2E, 4E)-N,N-Dimethyl-5-phenylpenta-2,4-dienamide* (**3o**). White solid; yield: 74%; m.p.: 108–113 °C. IR (KBr plate): *ν*_max_ 1643 (C=O), 1624 (C=C), 1125 (C-N), 812 (Ar-H), 758 (Ar-H). ^1^H NMR (600 MHz, CDCl_3_) δ 7.46–7.41 (m, 3H, Ar-CH=, Ar-H, -CH=), 7.33 (t, *J* = 7.6 Hz, 2H, Ar-H), 7.27 (d, *J* = 7.3 Hz, 1H, -CH=), 6.92–6.83 (m, 2H, Ar-H), 6.45 (d, *J* = 14.7 Hz, 1H, CO-CH=), 3.10 (s, 3H, CH_3_), 3.03 (s, 3H, CH_3_). ^13^C NMR (150 MHz, CDCl_3_) δ 166.8 (C=O), 142.5 (-CH=), 139.0 (-CH=), 136.4 (Ar-C), 128.8 (Ar-C), 128.7 (Ar-C), 127.0 (Ar-C), 126.9 (-CH=), 120.6 (-CH=), 37.4 (CH_3_), 35.9 (CH_3_). HRMS-ESI (*m*/*z*): calcd. for C_13_H_16_ON [M + H]^+^: 202.1226; found: 202.1224.

## 4. Conclusions

In conclusion, we have developed an efficient PPA-promoted direct aldol condensation of amides for the straightforward synthesis of cinnamides. A range of cinnamides have been prepared in moderate-to-good yields. Compared to previous approaches, the methodology was characterized by the use of equivalent amides and a short reaction time.

## Figures and Tables

**Table 1:** Optimization of reaction conditions *^a^*.

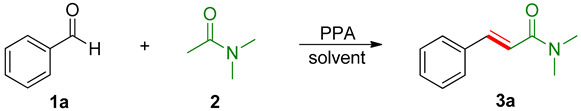					
Entry	PPA (Equiv.)	*T* (°C)	t (h)	Solvent	3a (%) *^b^*
1	2	reflux	4	DMF	46
2	1.5	reflux	4	DMF	45
3	1	reflux	4	DMF	50
4	0.5	reflux	4	DMF	22
5	1	140	4	DMF	13
6	1	reflux	5	DMF	64
7	1	reflux	6	DMF	79
8	1	reflux	7	DMF	77
9	1	reflux	6	1,4-Dioxane	6
10	1	reflux	6	THF	/
11	1	reflux	6	Toluene	15
12	1	reflux	6	DCE	/
13	1	reflux	6	DMSO	/
14	1	reflux	6	EtOH	/

*^a^* Reaction conditions: **1a** (1 mmol), **2** (1 mmol), and solvent (1 mL). The boiling point of DMF is 153 **°**C. *^b^* Isolated yield.

**Table 2:** Evaluation of substrate scope *^a^*.

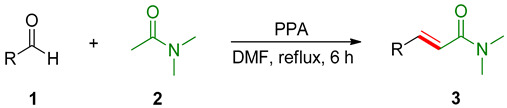
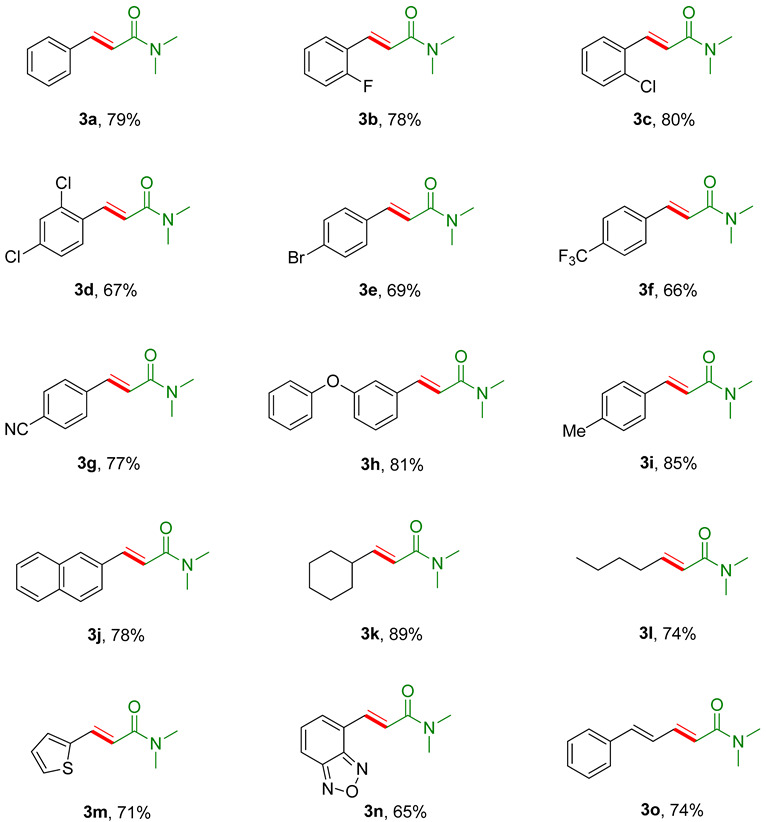

*^a^* Reaction conditions: 1 (1 mmol), 2 (1 mmol), PPA (1 mmol), and DMF (1 mL). Isolated yield.

## Data Availability

Data are contained within the article and Supplementary Materials.

## Supplementary Materials

The following supporting information can be downloaded at: https://www.mdpi.com/article/10.3390/molecules29194632/s1, copies of the ^1^H NMR and ^13^C NMR spectra for all compounds.

## References

[1] Harrold M.W., Wallace R.A., Farooqui T., Wallace L.J., Uretsky N., Miller D.D. (1989). Synthesis and D2 dopaminergic activity of pyrrolidinium, tetrahydrothiophenium, and tetrahydrothiophene analogs of sulpiride. J. Med. Chem..

[2] Pardin C., Pelletier J.N., Lubell W.D., Keillor J.W. (2008). Cinnamoyl inhibitors of tissue transglutaminase. J. Org. Chem..

[3] Kanemasa S., Yamamoto H., Kobayashi S. (1996). dl-Selective reductive coupling/dieckmann condensation sequence of α,β-unsaturated amides with samarium (II) iodide/HMPA. Synthesis of a new ligand, *trans*-1,2-cyclopentanediyl-2,2′-biphenol. Tetrahedron Lett..

[4] Nahm M.R., Potnick J.R., White P.S., Johnson J.S. (2006). Metallophosphite-catalyzed asymmetric acylation of α,β-unsaturated amides. J. Am. Chem. Soc..

[5] Nemoto T., Kakei H., Gnanadesikan V., Tosaki S.Y., Ohshima T., Shibasaki M. (2002). Catalytic asymmetric epoxidation of α,β-unsaturated amides: Efficient synthesis of β-aryl α-hydroxy amides using a one-pot tandem catalytic asymmetric epoxidation-pd-catalyzed epoxide opening process. J. Am. Chem. Soc..

[6] Lee J.E., Yun J. (2008). Catalytic asymmetric boration of acyclic α,β-unsaturated esters and nitriles. Angew. Chem. Int. Ed..

[7] Jiang X.F., Zhen Y.S. (2000). Cinnamamide, an antitumor agent with low cytotoxicity acting on matrix metalloproteinase. Anti-Cancer Drugs.

[8] De P., Baltas M., Bedos-Belval F. (2011). Cinnamic acid derivatives as anticancer agents-a review. Curr. Med. Chem..

[9] Deng X.Q., Wu D., Wei C.X., Quan Z.S. (2011). Synthesis and antidepressant-like action of *N*-(2-hydroxyethyl) cinnamamide derivatives in mice. Med. Chem. Res..

[10] Surendran S., Babu M., Joseph J., Padma U.D. (2020). Facilitatory effect of piperine on the anticonvulsant effect of sodium valproate against pentylenetetrazole induced seizures in mice. Res. J. Pharm. Technol..

[11] Wang Y., Gao M., Zhong Y., Wu B. (2023). Synthesis, crystal structure, hirshfeld surface analyses and biological activity of novel cinnamide derivatives as neuroprotective drugs. Polycycl. Aromat. Compd..

[12] Zhong Y., Xu Y., Tan X.Z., Wang Y.Y., Ma S.Y., Gao M.J., Wu B. (2024). Design, synthesis, and biological evaluation of novel cinnamide derivatives as neuroprotective agents for the treatment of cerebral ischemia. Curr. Med. Chem..

[13] Khan T.A., Al Nasr I.S., Koko W.S., Ma J., Eckert S., Brehm L., Said R.S.B., Daoud I., Hanachi R., Rahali S. (2023). Evaluation of the antiparasitic and antifungal activities of new synthetic piperlongumine-type cinnamide derivatives: Booster effect by halogen substituents. ChemMedChem.

[14] Valeur E., Bradley M. (2009). Amide bond formation: Beyond the myth of coupling reagents. Chem. Soc. Rev..

[15] Yamada K., Karuo Y., Tsukada Y., Kunishima M. (2016). Mild amide-cleavage reaction mediated by electrophilic benzylation. Chem.—Eur. J..

[16] Kim Y., Chang S. (2016). Borane-catalyzed reductive α-silylation of conjugated esters and amides leaving carbonyl groups intact. Angew. Chem. Int. Ed..

[17] Zhang J.R., Liao Y.Y., Deng J.C., Tang Z.L., Xu Y.L., Xu L., Tang R.Y. (2017). DABCO-Promoted decarboxylative acylation: Synthesis of α-keto and α,β-unsaturated amides or esters. Asian J. Org. Chem..

[18] Fujihara T., Katafuchi Y., Iwai T., Terao J., Tsuji Y. (2010). Palladium-catalyzed intermolecular addition of formamides to alkynes. J. Am. Chem. Soc..

[19] Kumar P.S., Kumar G.S., Kumar R.A., Reddy N.V., Rajender Reddy K. (2013). Copper-catalyzed oxidative coupling of carboxylic acids with *N*,*N*-dialkylformamides: An approach to the synthesis of amides. Eur. J. Org. Chem..

[20] Priyadarshini S., Joseph P.A., Kantam M.L. (2013). Copper catalyzed cross-coupling reactions of carboxylic acids: An expedient route to amides, 5-substituted γ-lactams and α-acyloxy esters. RSC Adv..

[21] Woodbury R.P., Rathke M.W. (1978). Formation of the lithium enolate of *N,N*-dimethyl-2-trimethylsilylacetamide. Reaction with carbonyl compounds and epoxides. J. Org. Chem..

[22] Böhm V.P., Herrmann W.A. (2000). Nonaqueous Ionic Liquids: Superior reaction media for the catalytic Heck-vinylation of chloroarenes. Chem.—Eur. J..

[23] Saberi D., Mahdudi S., Cheraghi S., Heydari A. (2014). Cu(II)-acetylacetone complex covalently anchored onto magnetic nanoparticles: Synthesis, characterization and catalytic evaluation in amide bond formation via oxidative coupling of carboxylic acids with *N*,*N*-dialkylformamides. J. Organomet. Chem..

[24] Yang X.H., Wei W.T., Li H.B., Song R.J., Li J.H. (2014). Oxidative coupling of alkenes with amides using peroxides: Selective amide C(sp^3^)-H *versus* C(sp^2^)-H functionalization. Chem. Commun..

[25] Lai M., Wu Z., Su F., Yu Y., Jing Y., Kong J., Wang Z., Wang S., Zhao M. (2020). Synthesis of cinnamides via amidation reaction of cinnamic acids with tetraalkylthiuram disulfides under simple condition. Eur. J. Org. Chem..

[26] Weidlich T., Prokeš L., Růžička A., Padělková Z. (2010). Condensation of aromatic aldehydes with *N*,*N*-dimethylacetamide in presence of dialkyl carbonates as dehydrating agents. Monatshefte Chem.-Chem. Mon..

[27] Foo S.W., Oishi S., Saito S. (2012). Aldol condensation of amides using phosphazene-based catalysis. Tetrahedron Lett..

[28] Yang T., Lu M., Lin Z., Huang M., Cai S. (2019). Visible-light-promoted oxidation/condensation of benzyl alcohols with dialkylacetamides to cinnamides. Org. Biomol. Chem..

[29] Liu H., Liu H., Wang E., Li L., Luo Z., Cao J., Chen J., Yang L., Yang X. (2023). Hydrogen bond assisted three-component tandem reactions to access *N*-alkyl-4-quinolones. Molecules.

[30] Wang E., Yang L., Yang Q., Yang F., Luo J., Gan M., Wang X., Song S., Lei Y., Yang X. (2022). Polyphosphoric acid-promoted one-pot synthesis and neuroprotective effects of flavanones against NMDA-induced injury in PC12 cells. RSC Adv..

[31] Yang L., Wang E., Fan Y., Yang J., Luo Z., Wang Y., Peng M., Deng T., Yang X. (2020). One-pot synthesis of (*E*)-3-benzylideneflavanones from 2-hydroxyacetophenones and aromatic aldehydes. Tetrahedron Lett..

[32] Yang Q., Wang Y., Yang J., Wu Y., Li L., Chen F., Wang E., Li L., Yang Y., Yan Y. (2021). Activation of phenolic oxygen atom using polyphosphoric acid: Synthesis of carbonyl-containing dihydrobenzofurans/dihydrobenzopyrans. Synth. Commun..

