# Supplementary material for: Polyphosphoric Acid-Promoted Efficient Synthesis of Cinnamides via Aldol Condensation of Amide
Source: Molecules. 2024 Sep 29;29(19):4632. doi: 10.3390/molecules29194632 (PMC11477491; doi:10.3390/molecules29194632)
Supplement: Supplementary file 1 [file molecules-29-04632-s001.zip › molecules-3208212-supplementary.pdf]

Supporting Information for

Polyphosphoric Acid-Promoted Efficient Synthesis of Cinnamides via Aldol Condensation of Amide

Enhua Wang [1,†], Lishou Yang [2,3,†], Lanfeng He [2,3], Qian Yang [1], Xue Wang [1], Yunlu Liu [1], Manxiang Li [1], Yang Lei [1,*] and Xiaosheng Yang [2,3,*]

1 Department of Food and Medicine, Guizhou Vocational College of Agriculture, Qingzhen 551400, China; enhuawang2023@sina.cn (E.W.); yangqian121212@sina.cn (Q.Y.); wangxue155@sina.com (X.W.); liuyunlu12@sina.com (Y.L.); limanxiang1224@sina.com (M.L.)

2 State Key Laboratory of Functions and Applications of Medicinal Plants, Guizhou Medical University, Guiyang 550014, China; lishouyang2023@sina.cn (L.Y.); yao320917547@sina.com (L.H.)

3 Natural Products Research Center of Guizhou Province, Guiyang 550014, China

* Correspondence: 123leiyang@sina.com (Y.L.); gzcnp@sina.cn (X.Y.)

† The authors contributed equally to this work.

1. General Information………………………………………………………..…………2

2. General Procedure for the Synthesis of Cinnamamides……………….…………2

3. ^{1}H NMR & ^{13}C NMR Spectra of the Products……………………………………...3

1. General Information

Unless otherwise noted, all reagents, catalysts and solvents were purchased from commercial suppliers and used without further purification. Column Chromatography was performed with silica gel (200-300 mesh). Melting points were determined using a X-4 melting point apparatus with microscope. The IR spectra were recorded with Mattson FTIR spectrometer 5000. Absorption maxima were measured in cm^{-1}. ^{1}H and ^{13}C NMR spectra were achieved on a Bruker AVANCE 600 MHz spectrometer (^{1}H 600 MHz; ^{13}C 150 MHz) in $CDCl_3$. High-resolution mass spectra were measured on a ThermoFish QE Focus facility. Thin-layer chromatographies were done on pre-coated silica gel 60F254 plates (Merck).

2. General Procedure for the Synthesis of Cinnamamides (3a-3o)

Benzaldehyde (1 mmol), DMA (1 mmol) and PPA (1 mmol) were added to a 5 mL round-bottomed flask containing DMF (1 mL) solvent. Under the protection of nitrogen, the reaction mixture was refluxed for 6 h. The reaction liquid was quenched with water and extracted with ethyl acetate. The organic layer was dried by anhydrous Na_2SO_4 and evaporated under reduced pressure. The resulting crude compound was purified by silica gel column chromatography to yield the pure products (**3a-3o**).

3. ^{1}H NMR & ^{13}C NMR Spectra of the Products

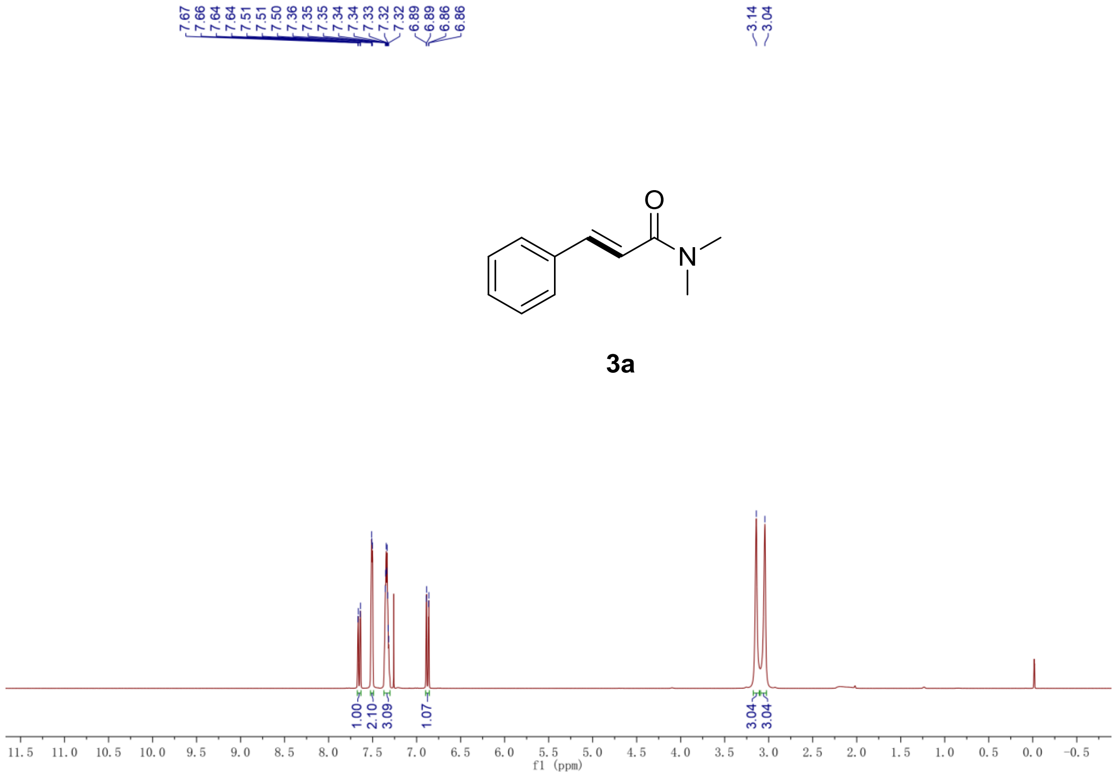

Figure S1. ^{1}H NMR (600 MHz, $CDCl_3$) spectrum of **3a**.

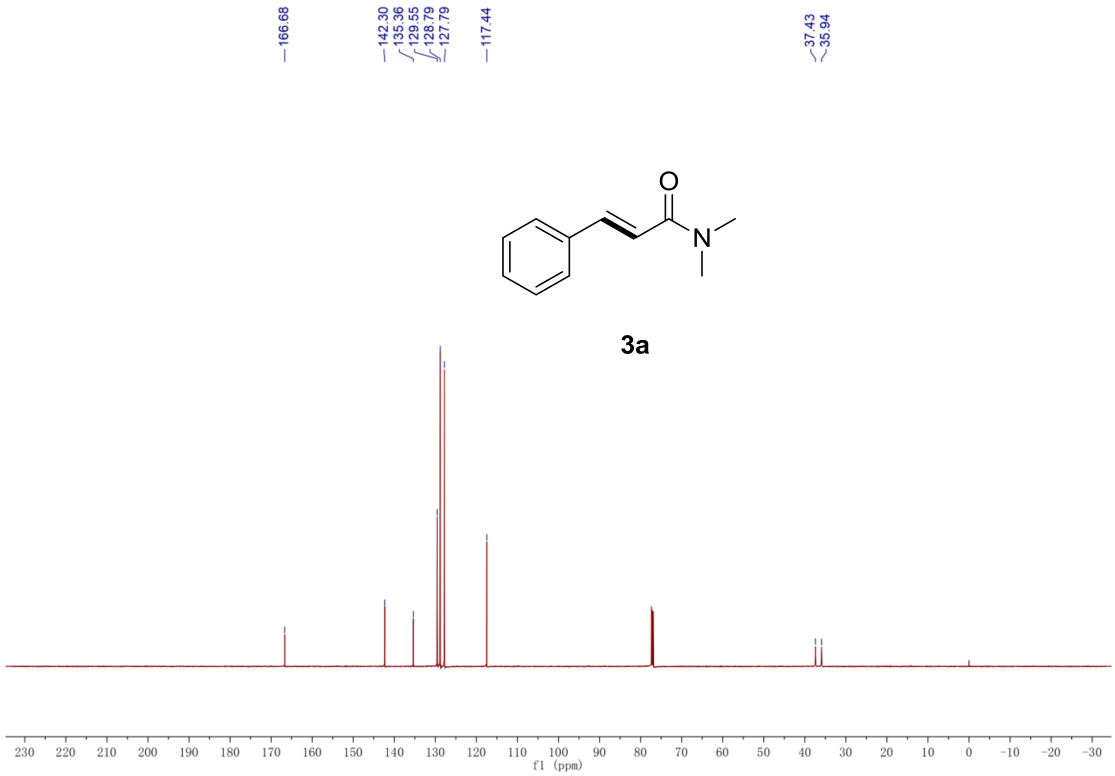

Figure S2. ^{13}C NMR (150 MHz, $CDCl_3$) spectrum of **3a**.

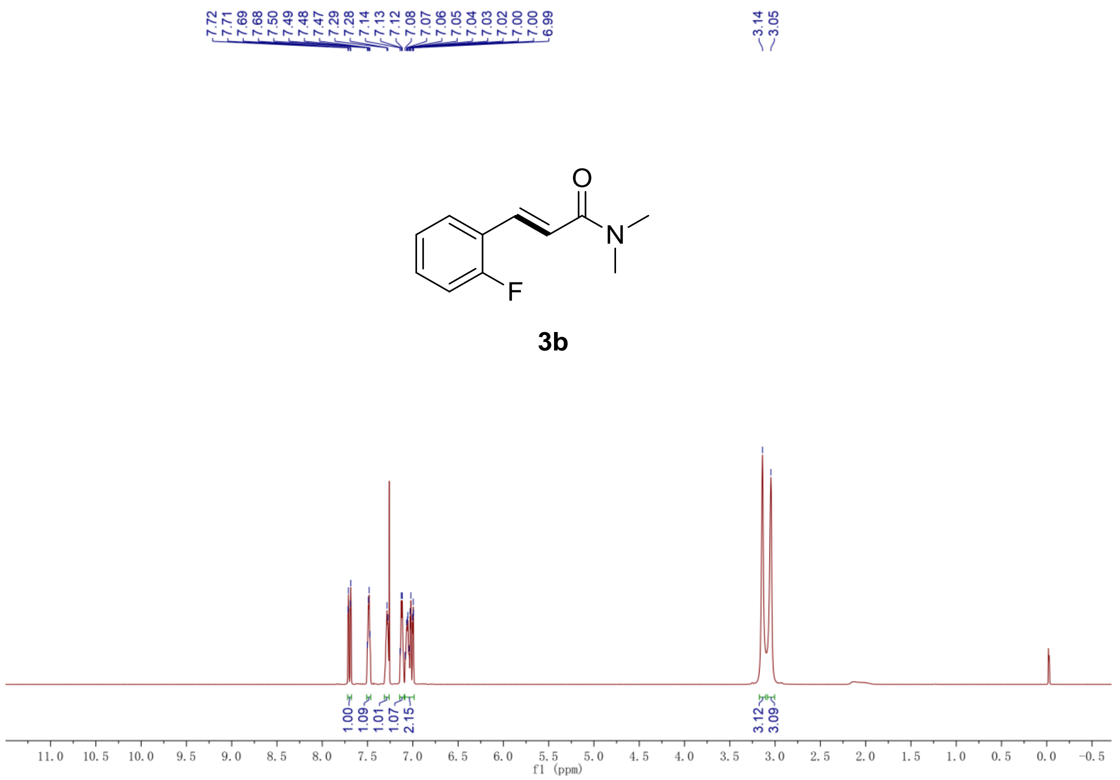

Figure S3. ^{1}H NMR (600 MHz, $CDCl_3$) spectrum of **3b**.

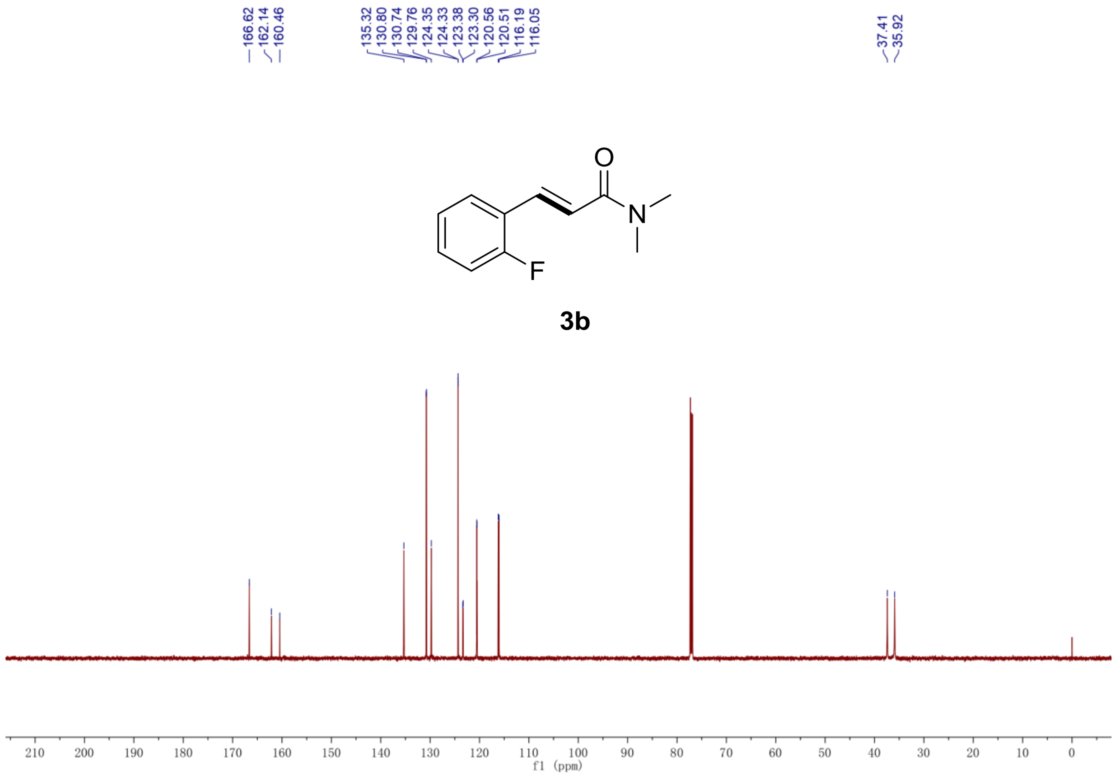

Figure S4. ^{13}C NMR (150 MHz, $CDCl_3$) spectrum of **3b**.

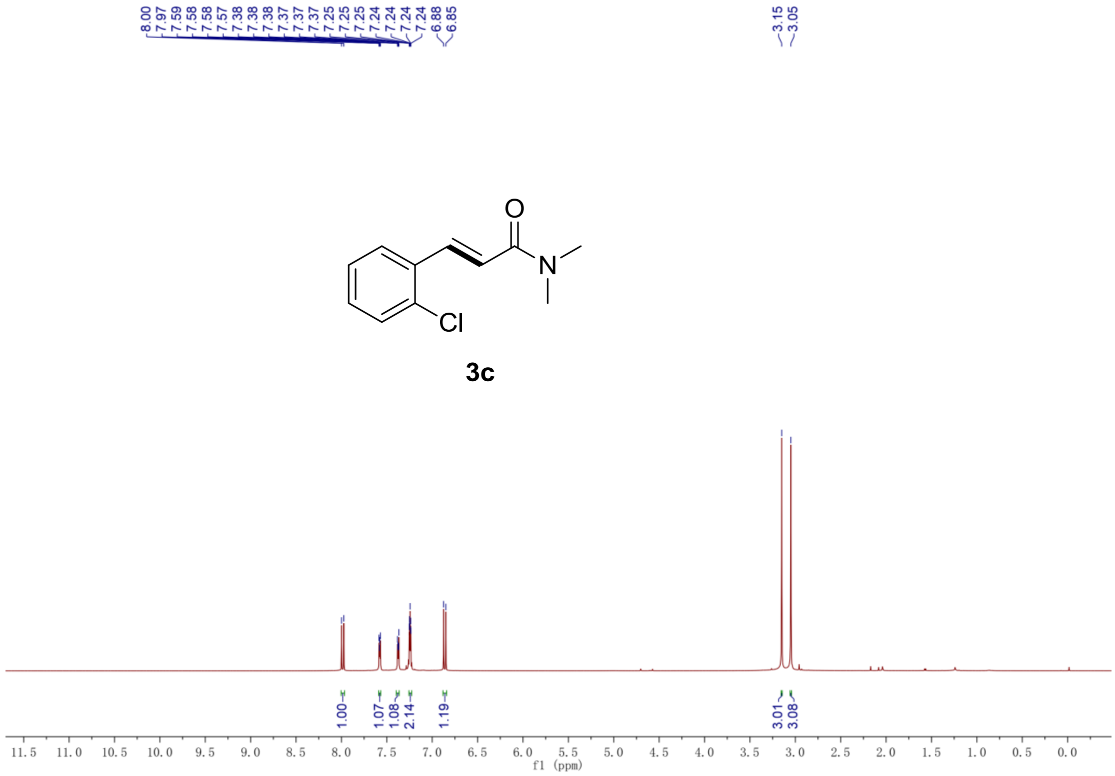

Figure S5. ^{1}H NMR (600 MHz, $CDCl_3$) spectrum of **3c**.

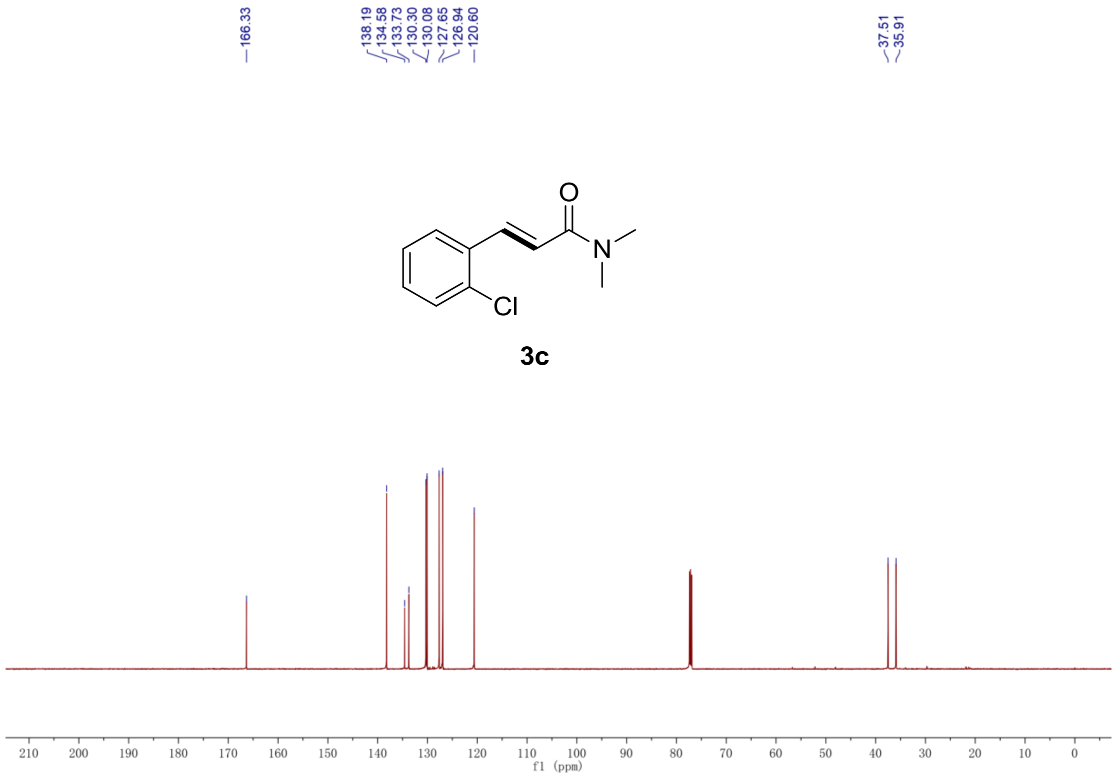

Figure S6. ^{13}C NMR (150 MHz, $CDCl_3$) spectrum of **3c**.

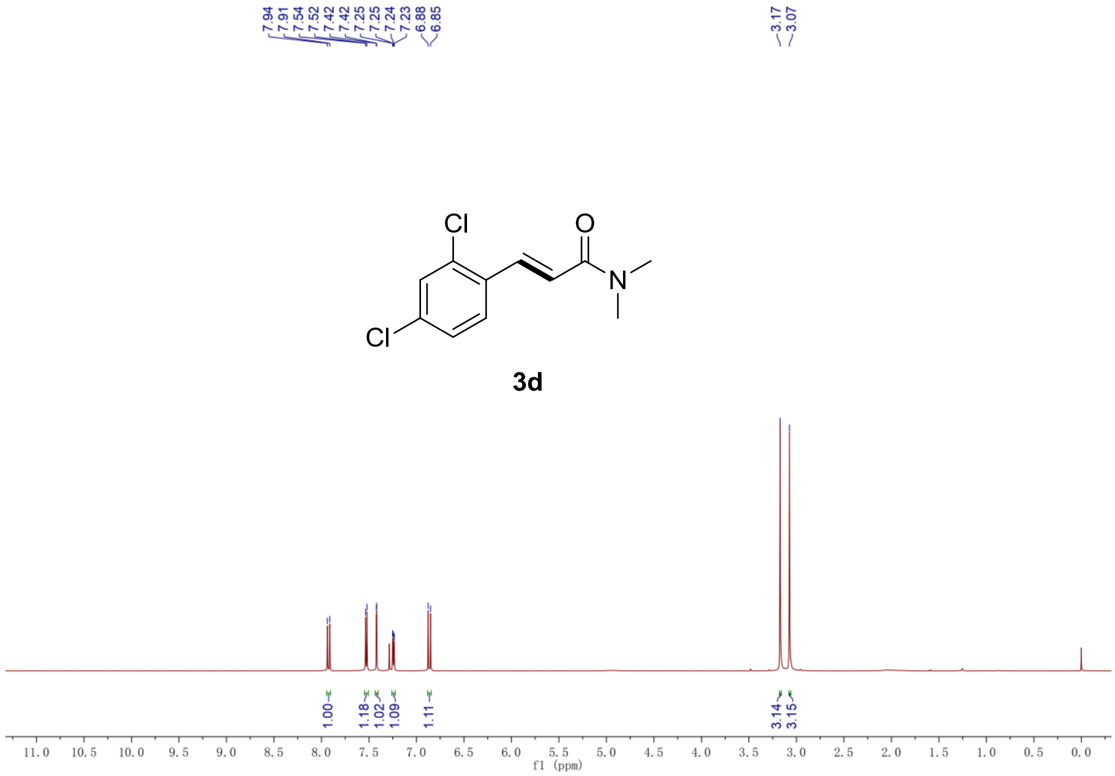

Figure S7. ^{1}H NMR (600 MHz, $CDCl_3$) spectrum of **3d**.

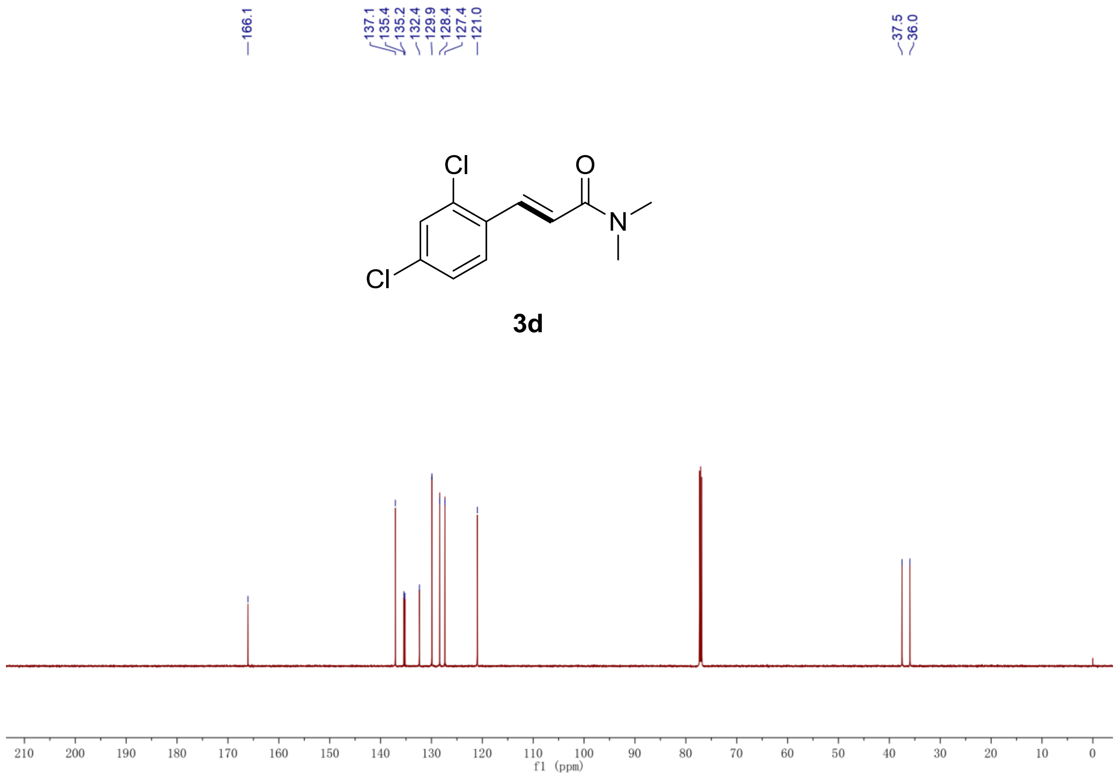

Figure S8. ^{13}C NMR (150 MHz, $CDCl_3$) spectrum of **3d**.

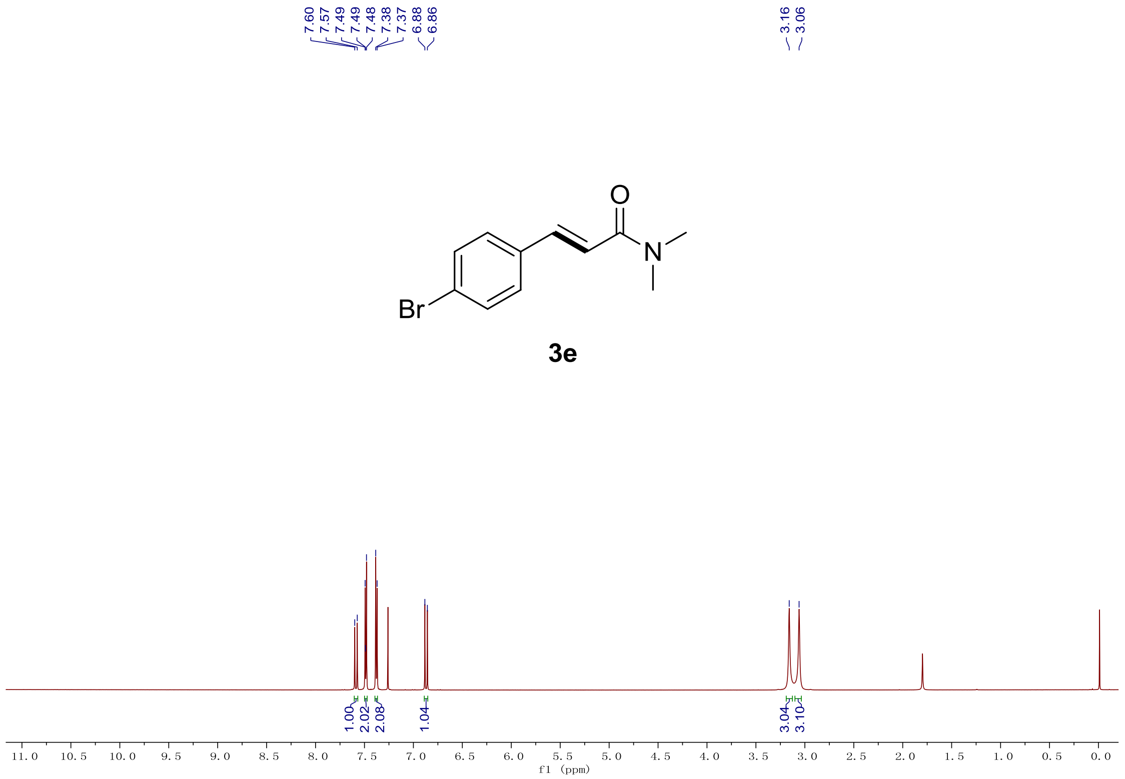

Figure S9. ^{1}H NMR (600 MHz, $CDCl_3$) spectrum of **3e**.

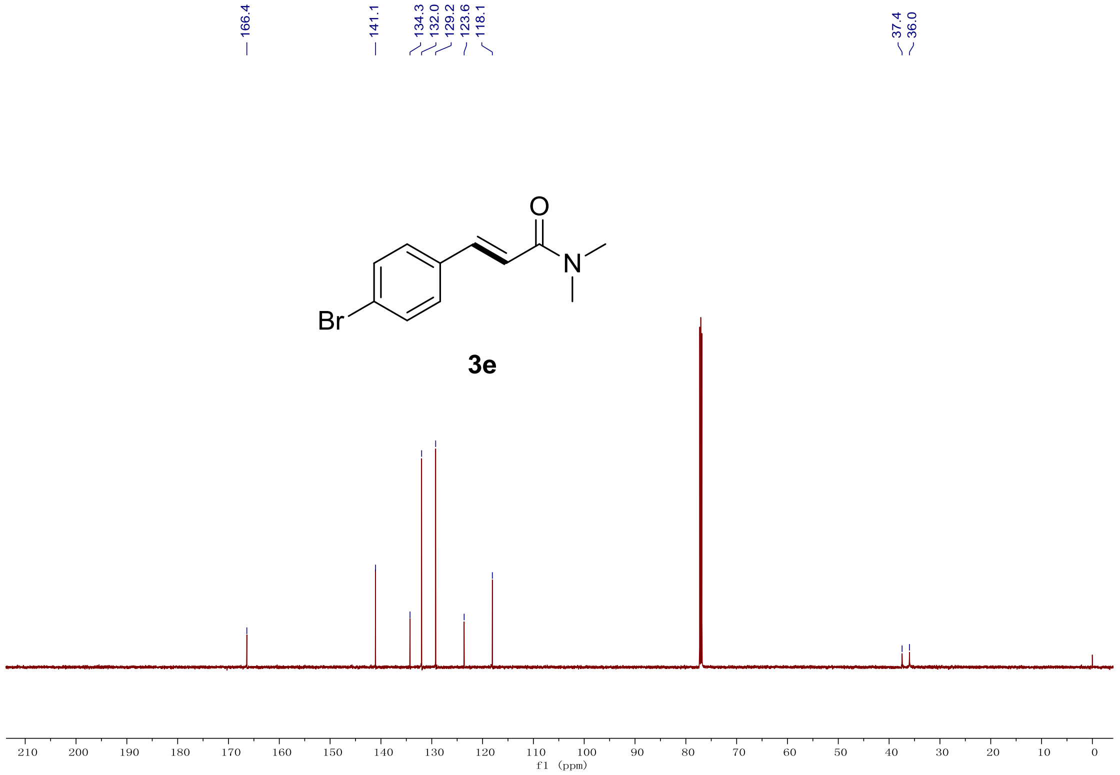

Figure S10. ^{13}C NMR (150 MHz, $CDCl_3$) spectrum of **3e**.

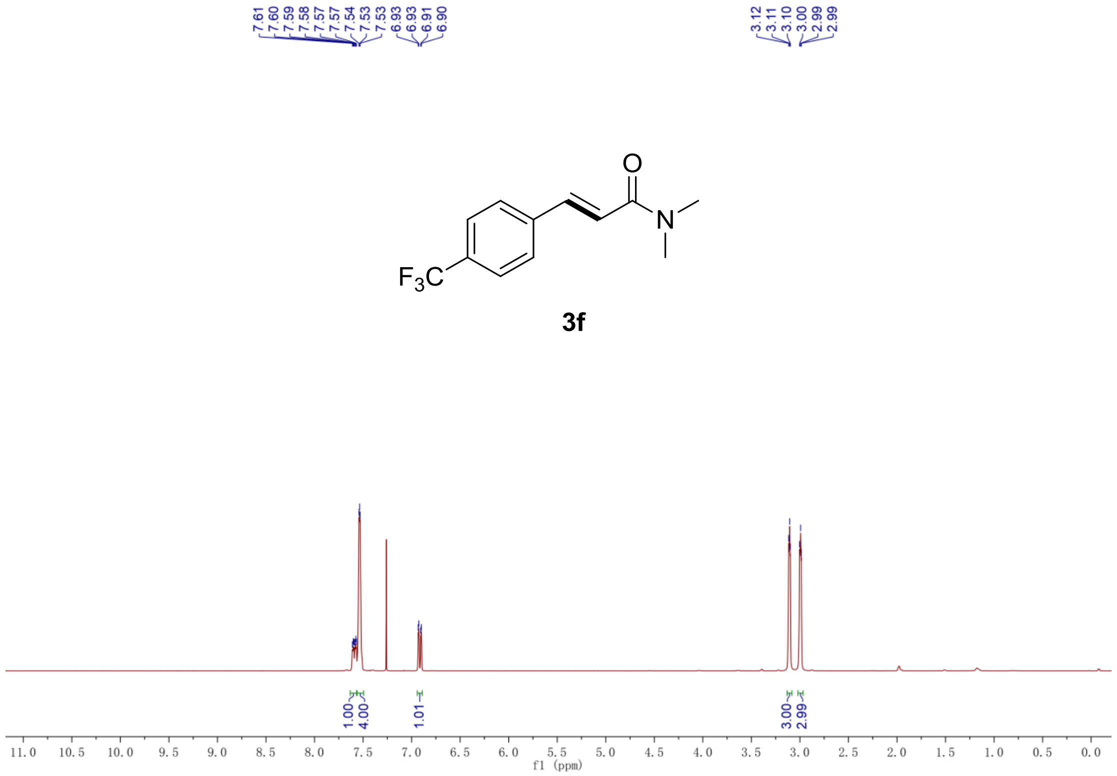

Figure S11. ^{1}H NMR (600 MHz, $CDCl_3$) spectrum of **3f**.

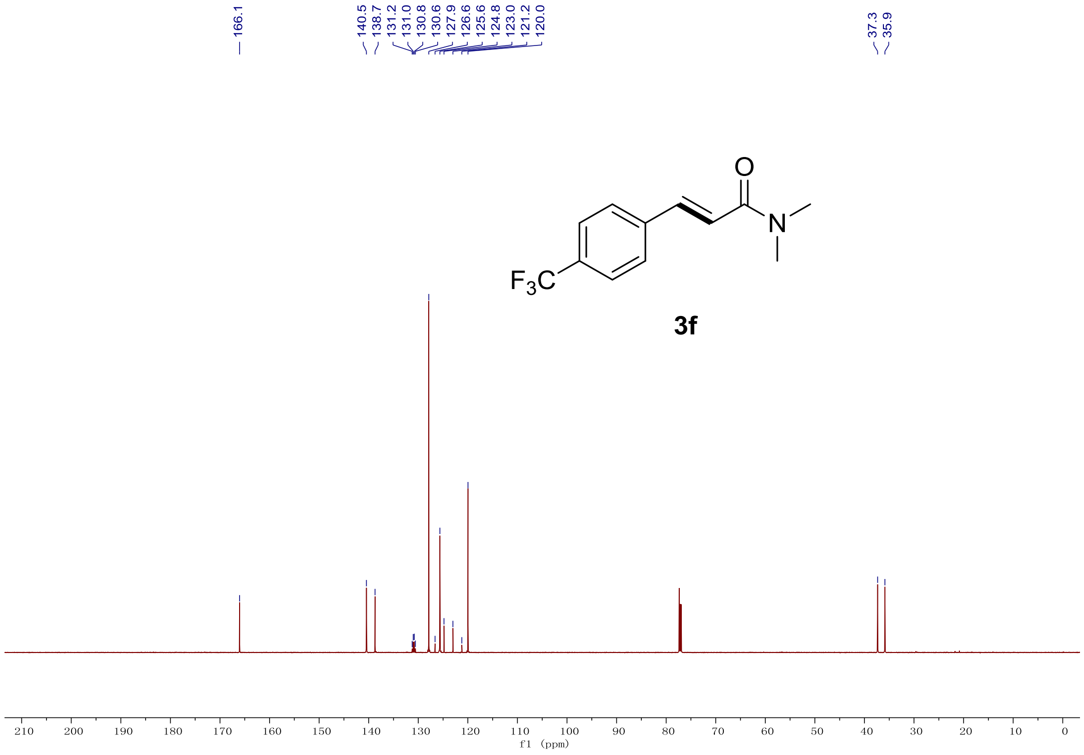

Figure S12. ^{13}C NMR (150 MHz, $CDCl_3$) spectrum of **3f**.

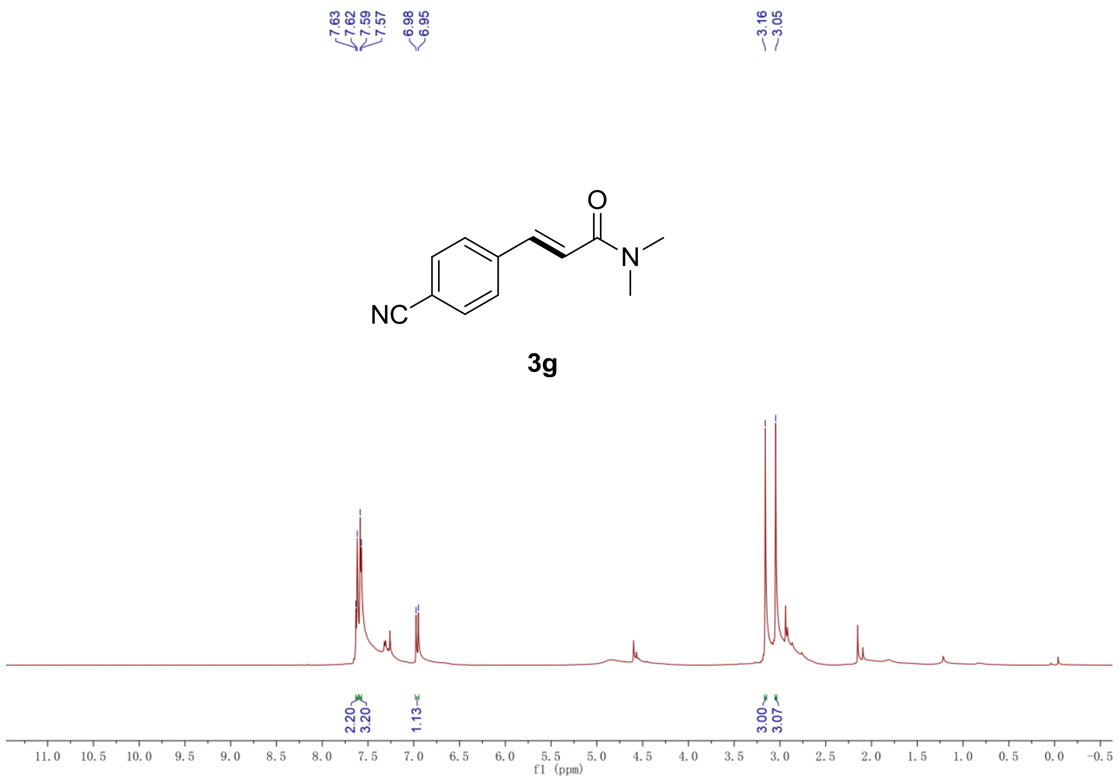

Figure S13. ^{1}H NMR (600 MHz, $CDCl_3$) spectrum of **3g**.

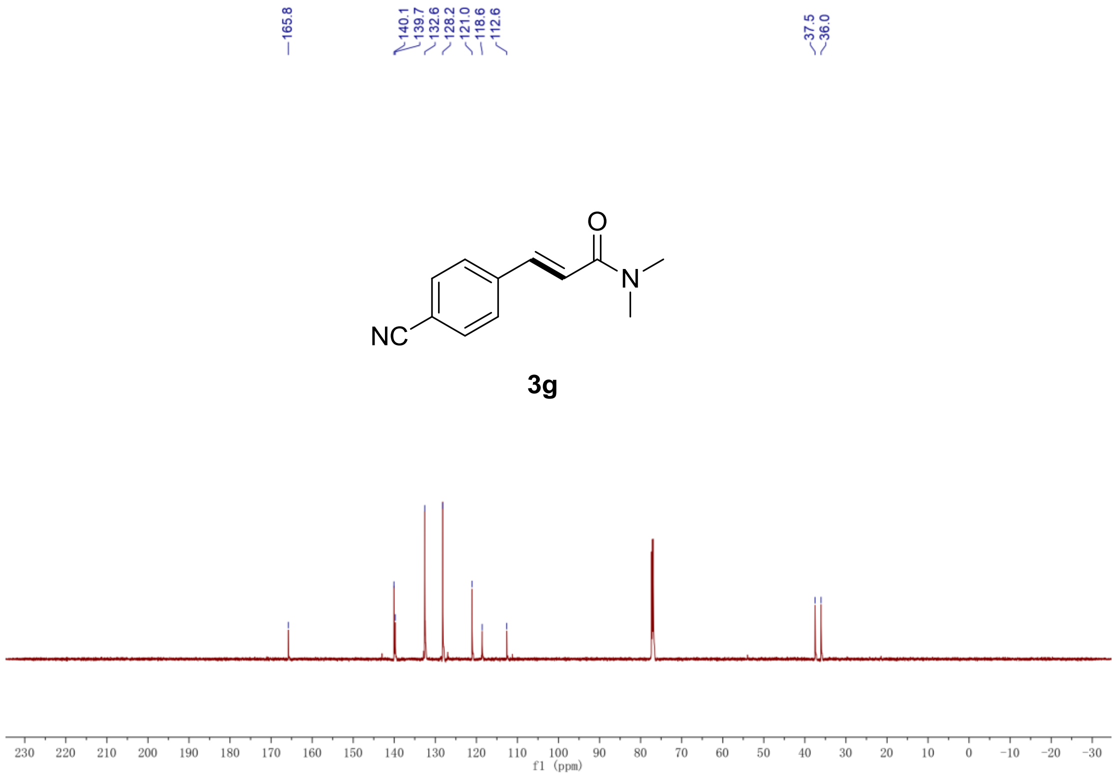

Figure S14. ^{13}C NMR (150 MHz, $CDCl_3$) spectrum of **3g**.

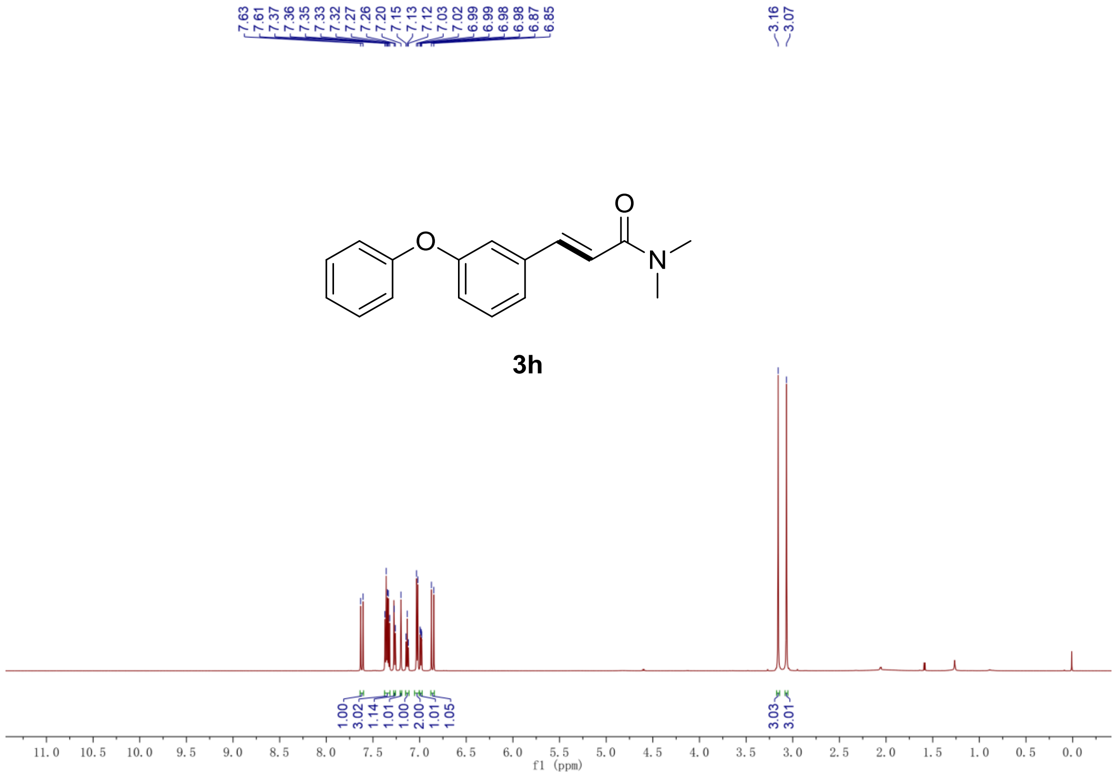

Figure S15. ^{1}H NMR (600 MHz, $CDCl_3$) spectrum of **3h**.

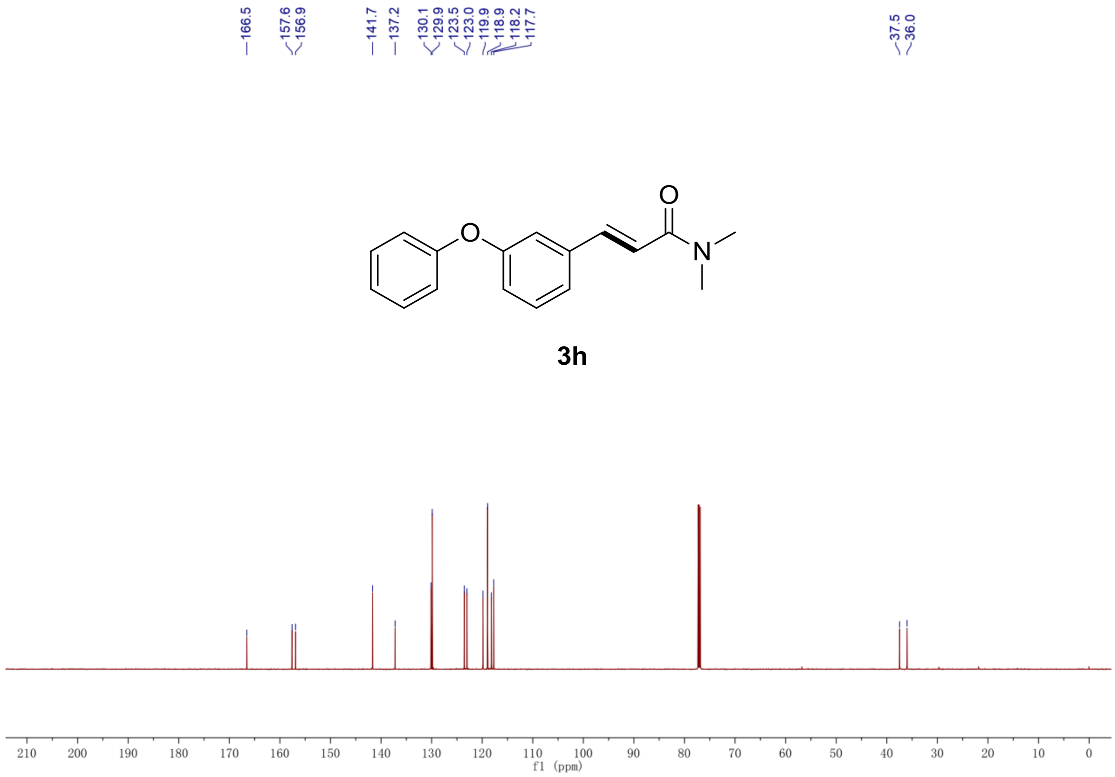

Figure S16. ^{13}C NMR (150 MHz, $CDCl_3$) spectrum of **3h**.

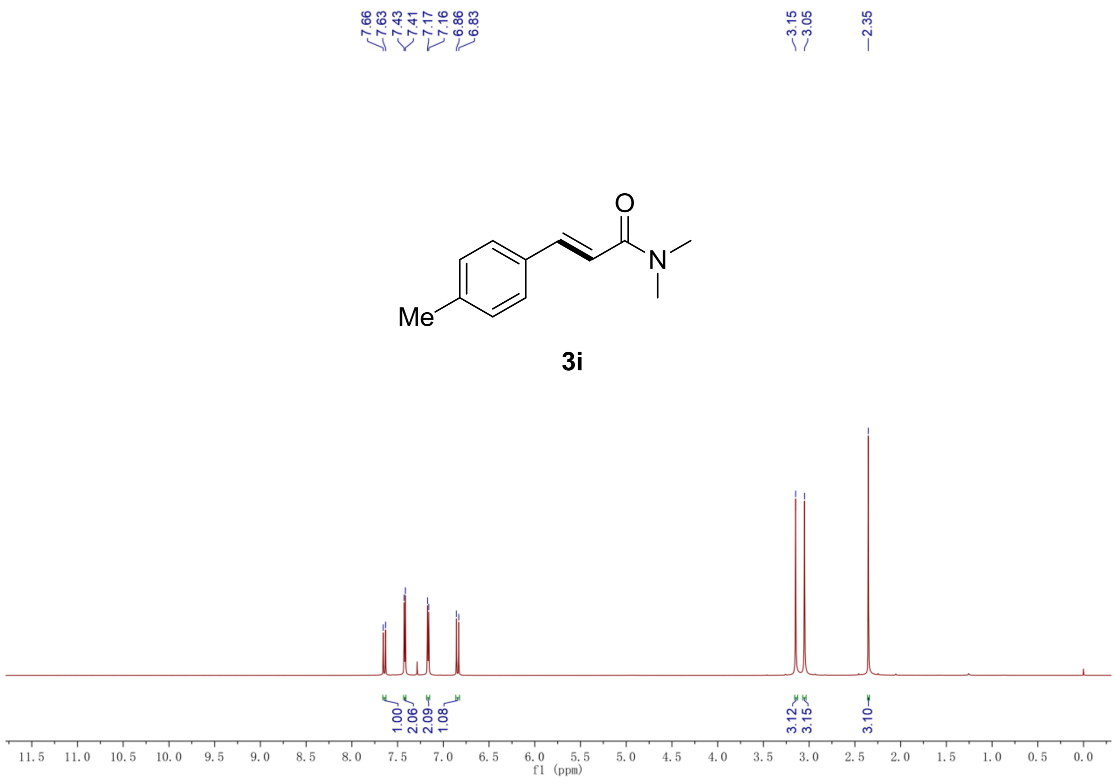

Figure S17. ^{1}H NMR (600 MHz, $CDCl_3$) spectrum of **3i**.

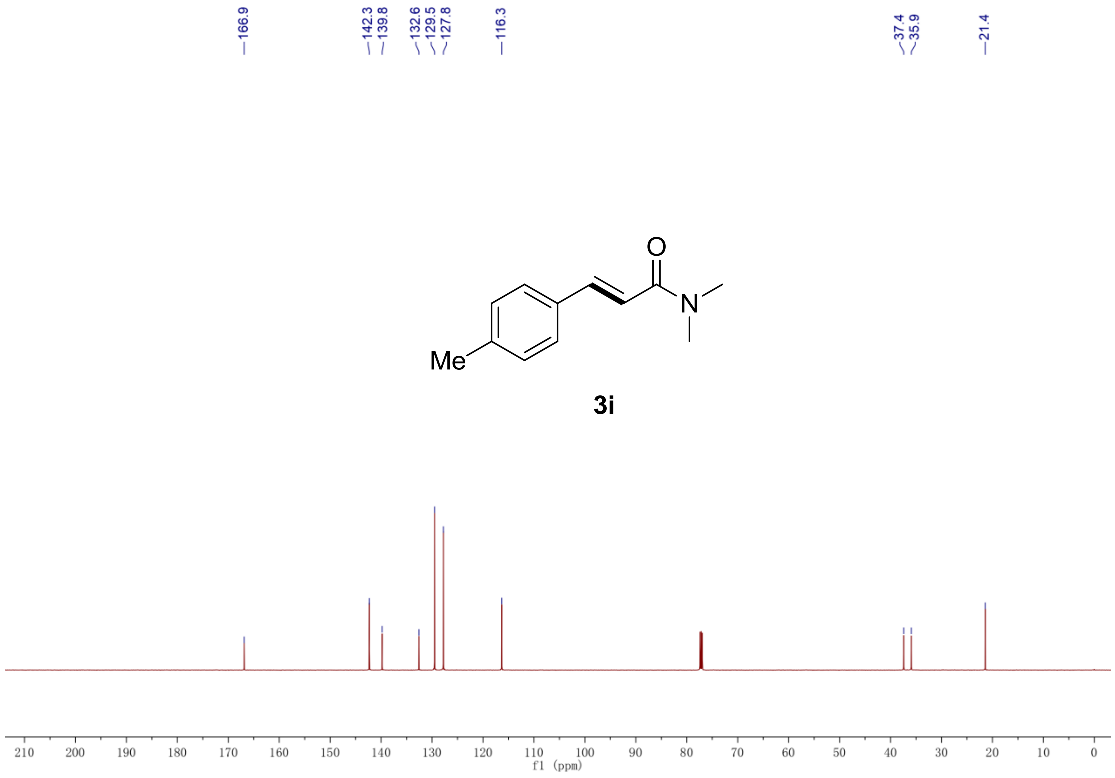

Figure S18. ^{13}C NMR (150 MHz, $CDCl_3$) spectrum of **3i**.

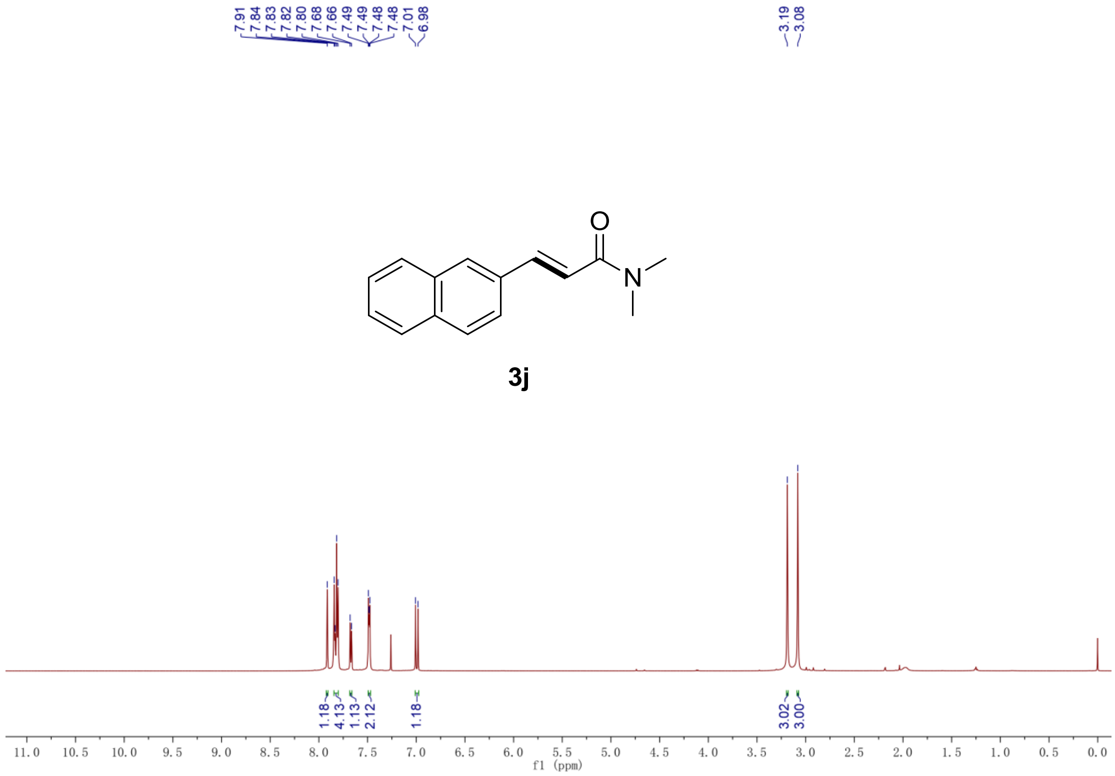

Figure S19. ^{1}H NMR (600 MHz, $CDCl_3$) spectrum of **3j**.

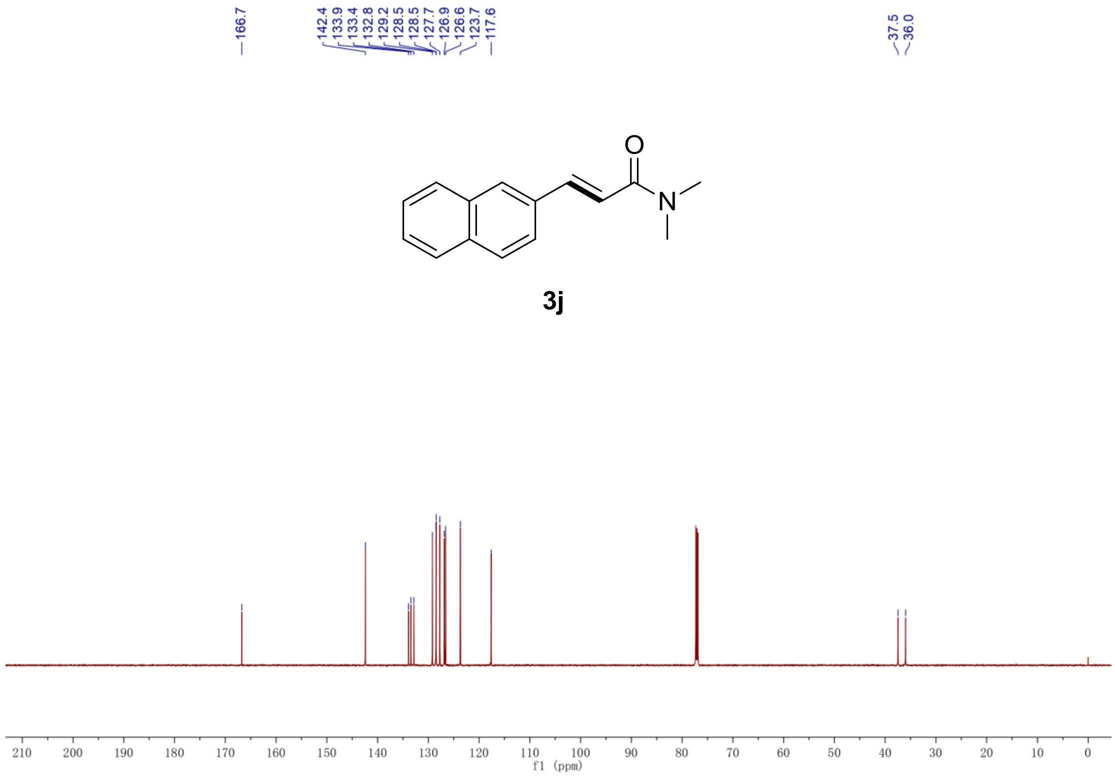

Figure S20. ^{13}C NMR (150 MHz, $CDCl_3$) spectrum of **3j**.

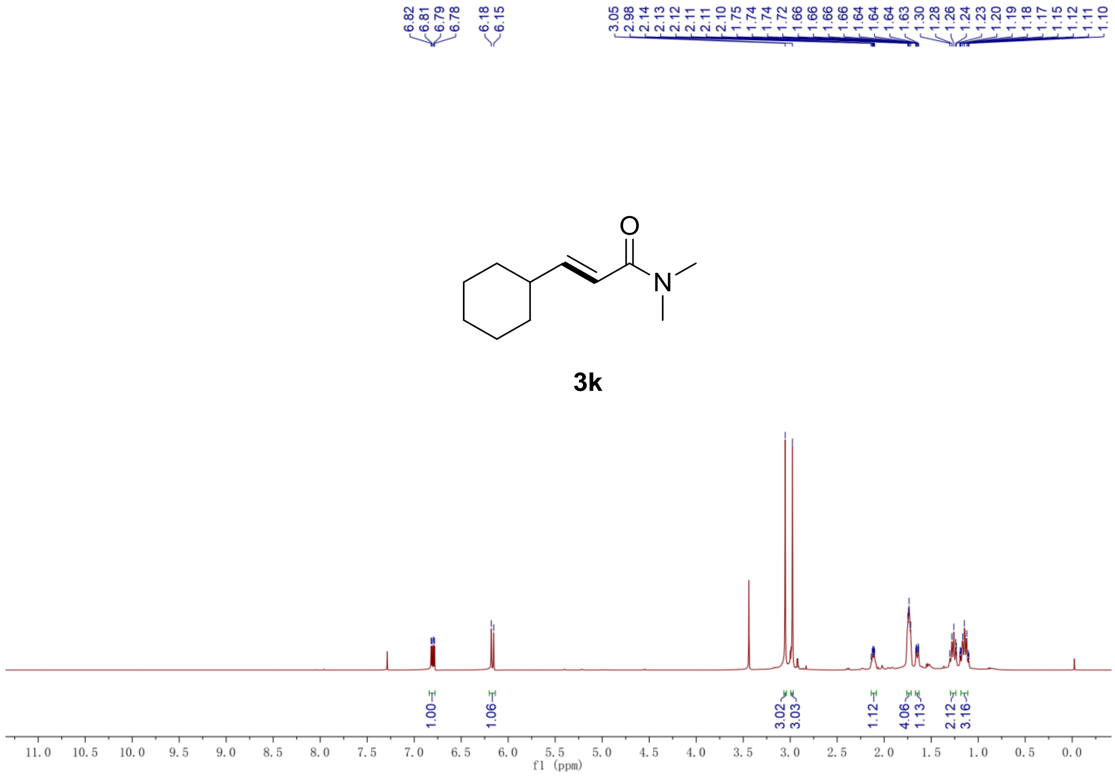

Figure S21. ^{1}H NMR (600 MHz, $CDCl_3$) spectrum of **3k**.

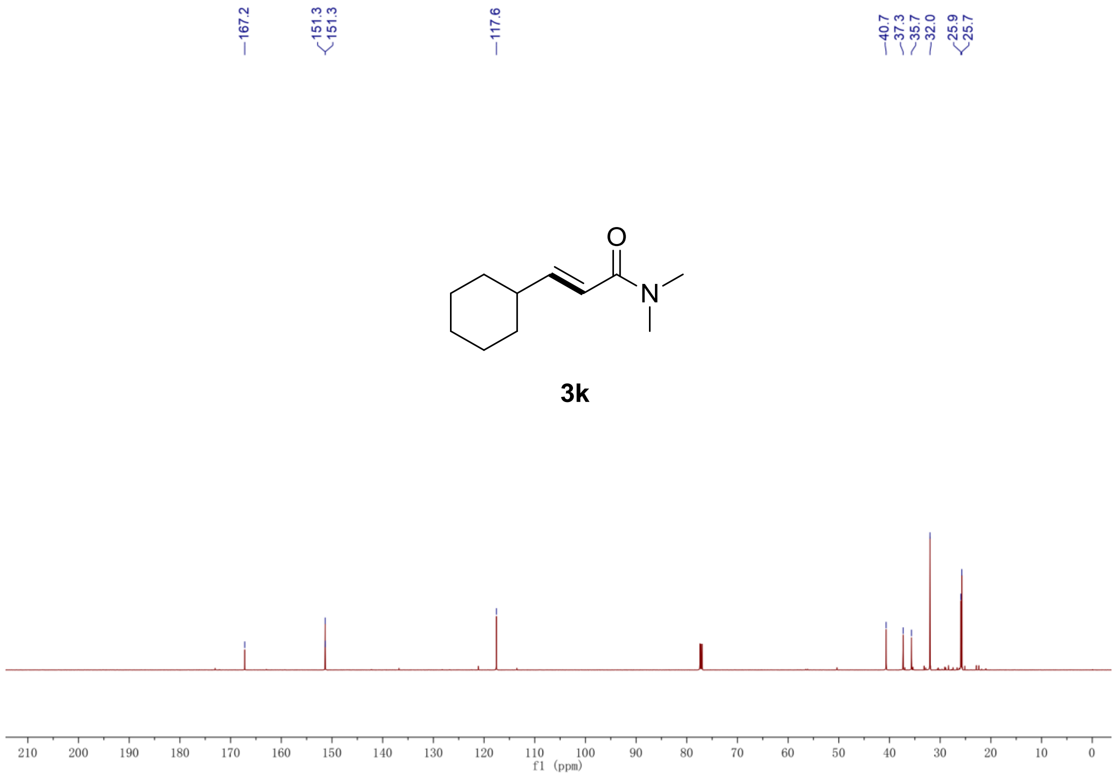

Figure S22. ^{13}C NMR (150 MHz, $CDCl_3$) spectrum of **3k**.

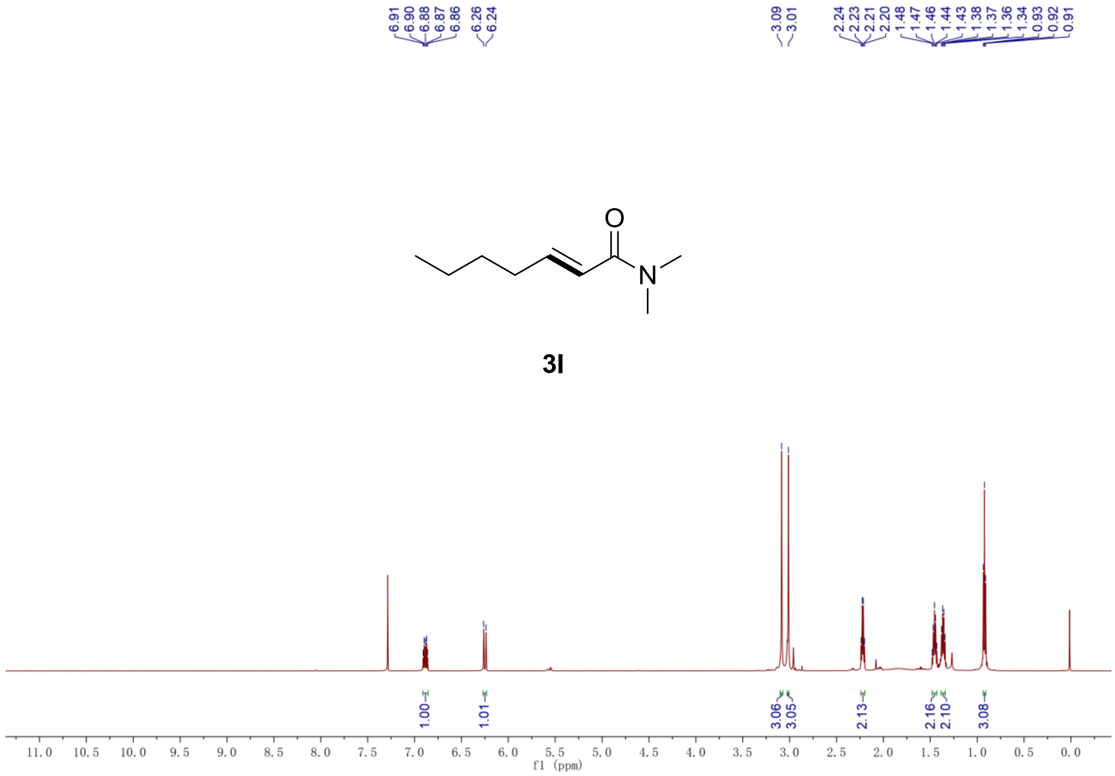

Figure S23. ^{1}H NMR (600 MHz, $CDCl_3$) spectrum of **3l**.

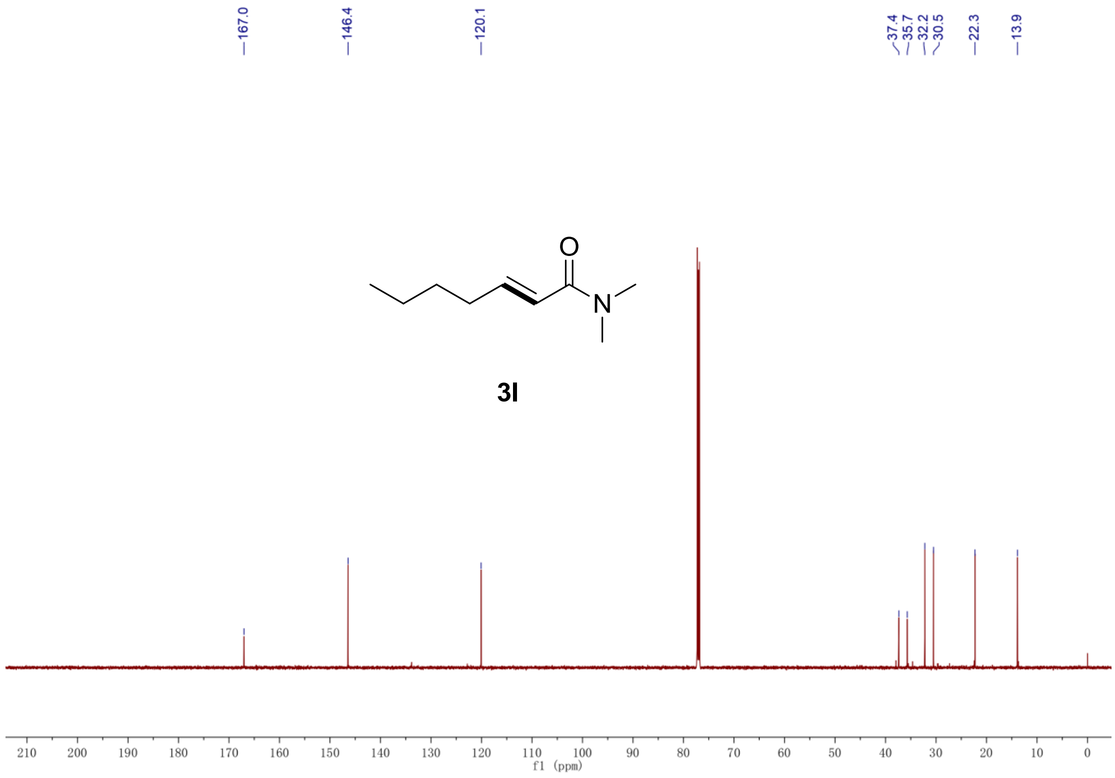

Figure S24. ^{13}C NMR (150 MHz, $CDCl_3$) spectrum of **3l**.

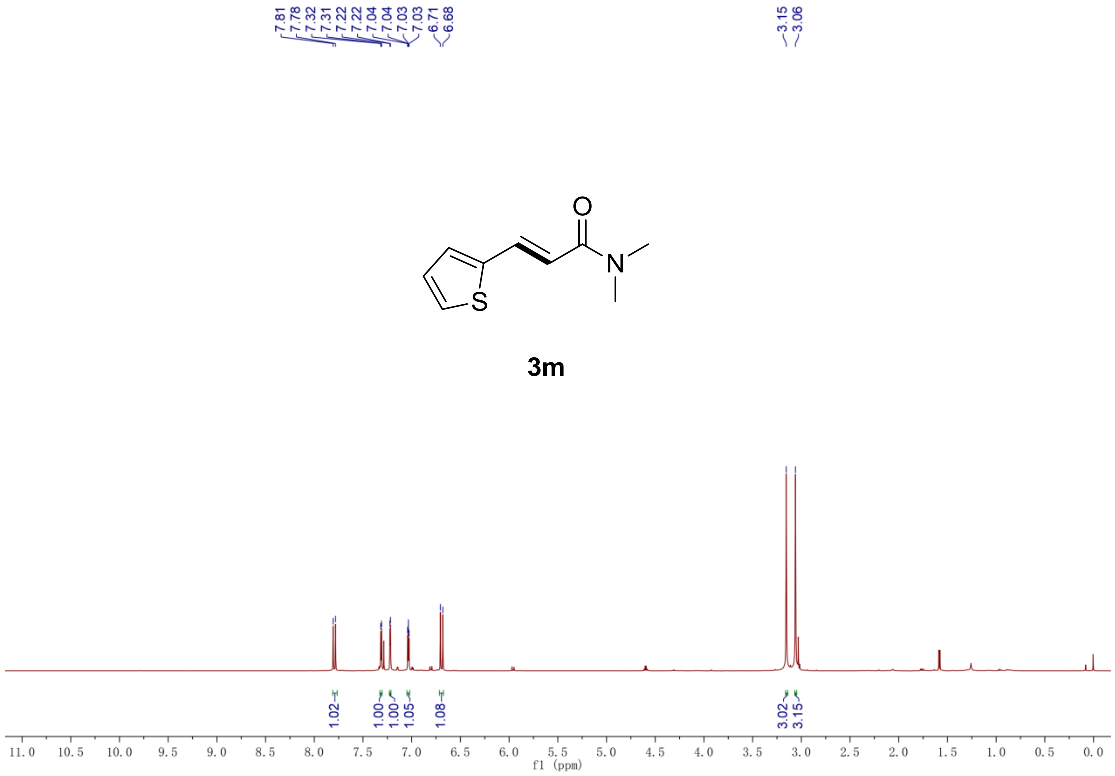

Figure S25. ^{1}H NMR (600 MHz, $CDCl_3$) spectrum of **3m**.

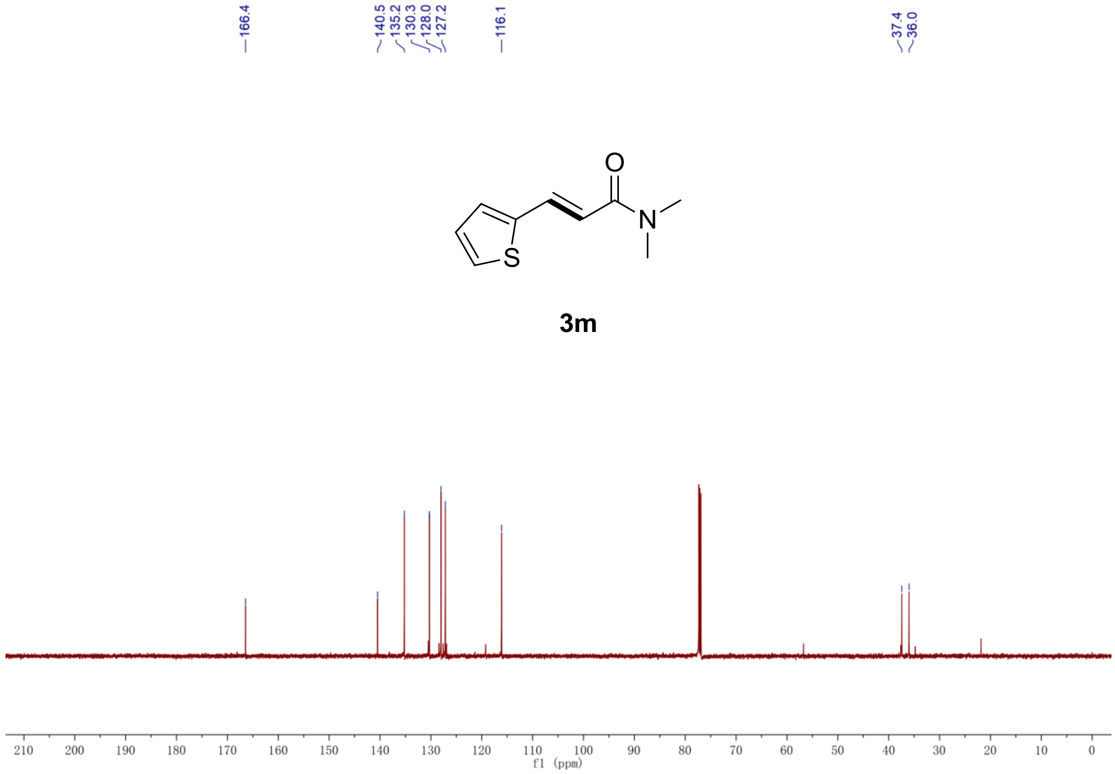

Figure S26. ^{13}C NMR (150 MHz, $CDCl_3$) spectrum of **3m**.

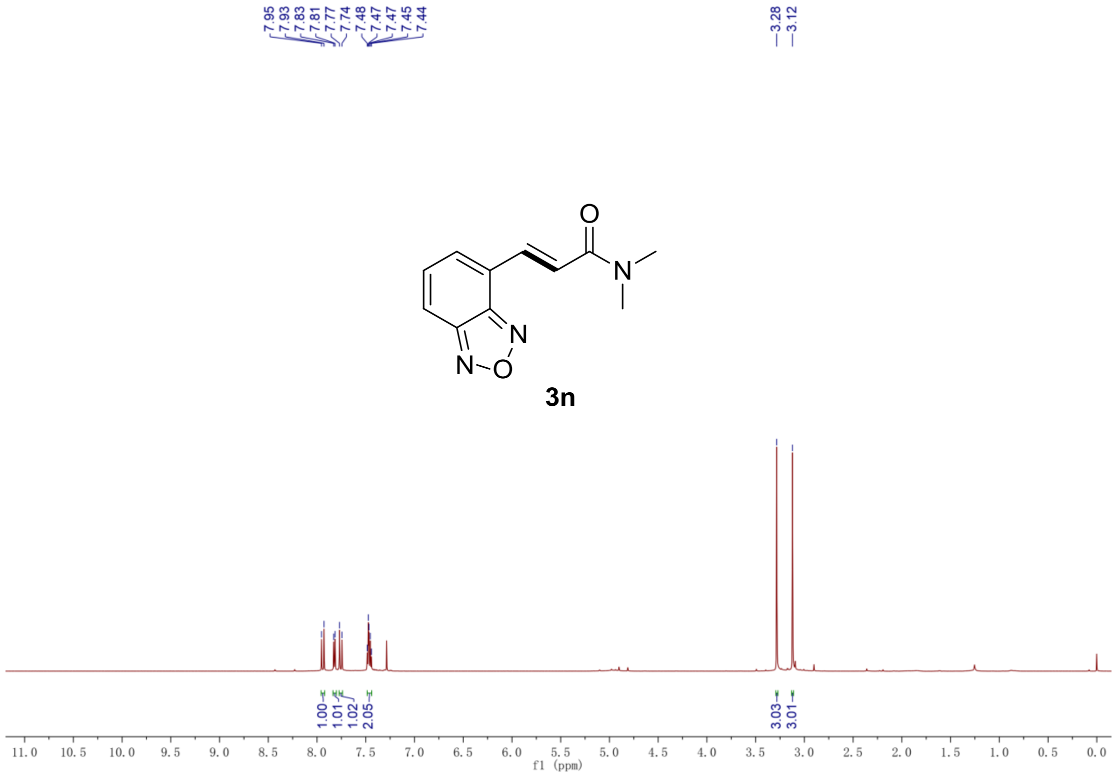

Figure S27. ^{1}H NMR (600 MHz, $CDCl_3$) spectrum of **3n**.

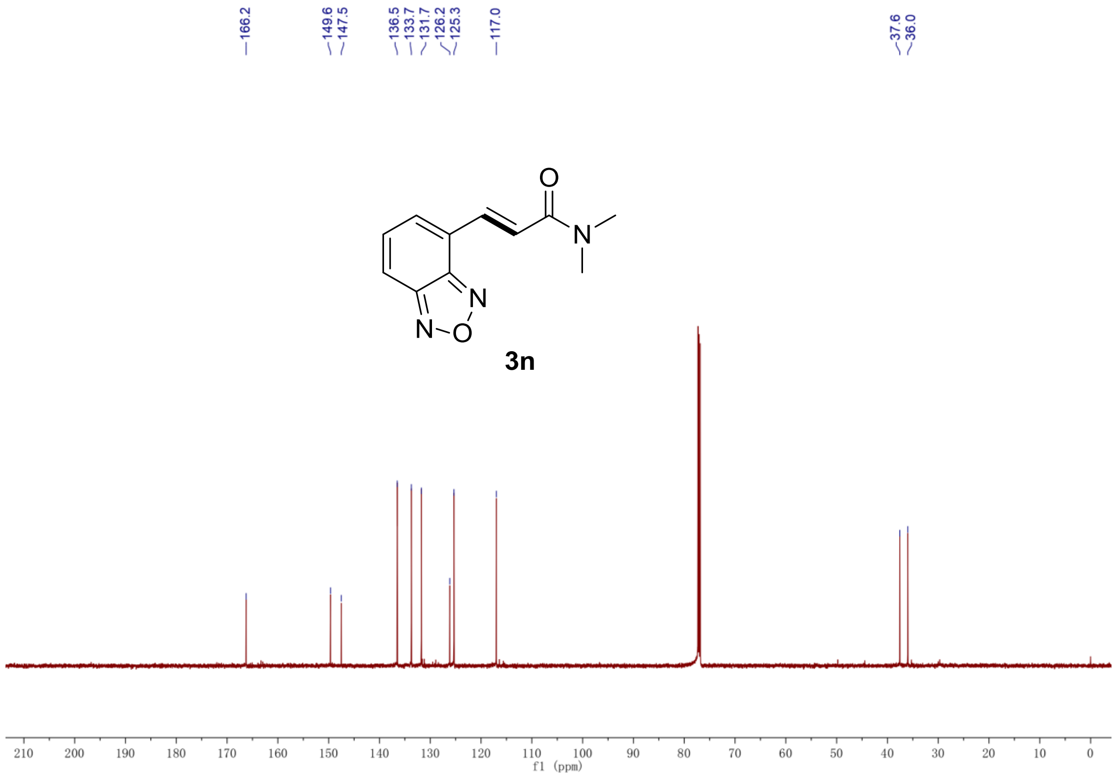

Figure S28. ^{13}C NMR (150 MHz, $CDCl_3$) spectrum of **3n**.

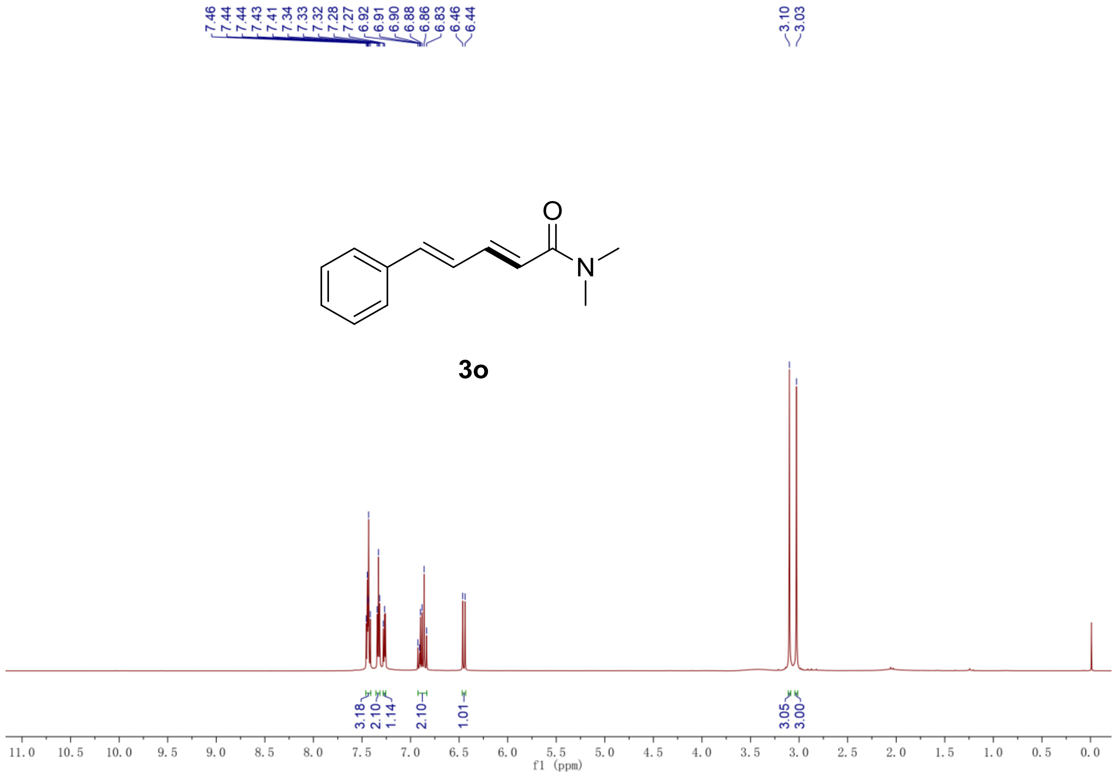

Figure S29. ^{1}H NMR (600 MHz, $CDCl_3$) spectrum of **3o**.

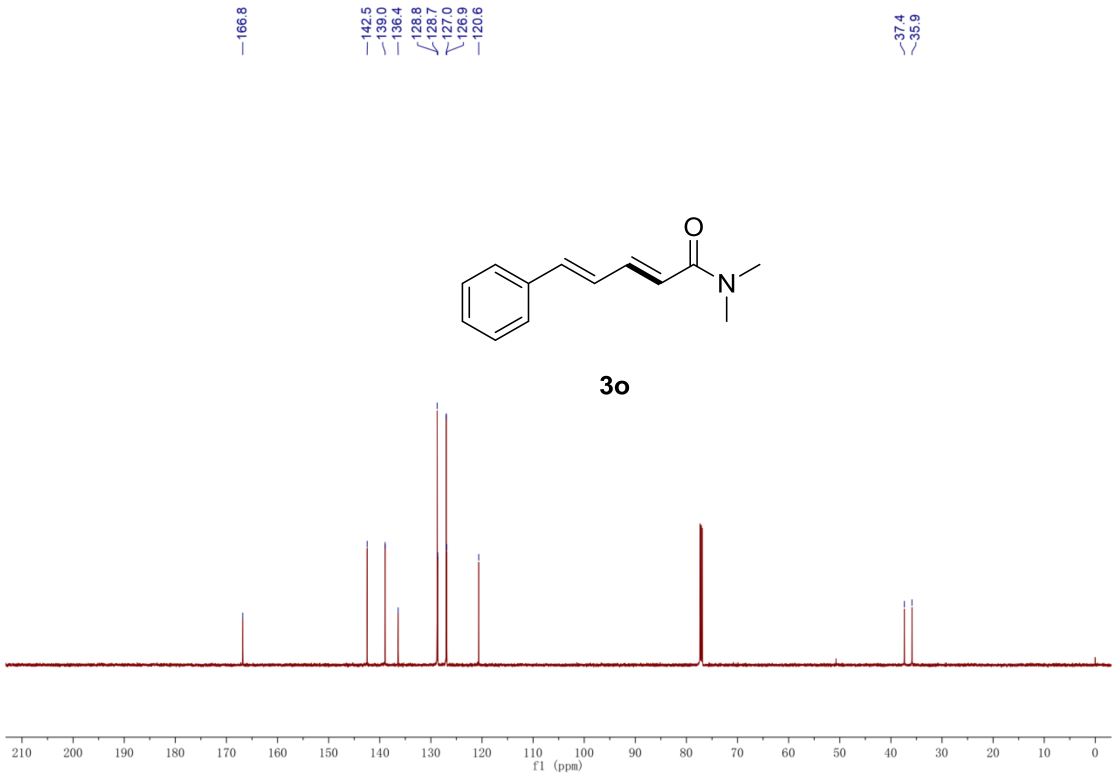

Figure S30. ^{13}C NMR (150 MHz, $CDCl_3$) spectrum of **3o**.
